# Autophosphorylation of the Tousled-like kinases TLK1 and TLK2 regulates recruitment to damaged chromatin via PCNA interaction

**DOI:** 10.1093/nar/gkae1279

**Published:** 2024-12-27

**Authors:** Kirk L West, Tram T N Nguyen, Kyle A Tengler, Natasha Kreiling, Kevin D Raney, Gargi Ghosal, Justin W Leung

**Affiliations:** Department of Radiation Oncology, University of Arkansas for Medical Sciences, 4301 Markham St, Little Rock, AR 72205, USA; Department of Biochemistry and Molecular Biology, University of Arkansas for Medical Sciences, 4301 Markham St, Little Rock, AR 72205, USA; Department of Radiation Oncology, University of Texas Health and Science Center, 7703 Floyd Curl Dr, San Antonio, TX 78229, USA; Department of Radiation Oncology, University of Texas Health and Science Center, 7703 Floyd Curl Dr, San Antonio, TX 78229, USA; Department of Genetics, Cell Biology and Anatomy, University of Nebraska Medical Center, S 42nd &, Emile St, Omaha, NE 68198, USA; Department of Biochemistry and Molecular Biology, University of Arkansas for Medical Sciences, 4301 Markham St, Little Rock, AR 72205, USA; Department of Genetics, Cell Biology and Anatomy, University of Nebraska Medical Center, S 42nd &, Emile St, Omaha, NE 68198, USA; Department of Radiation Oncology, University of Texas Health and Science Center, 7703 Floyd Curl Dr, San Antonio, TX 78229, USA

## Abstract

Tousled-like kinases 1 and 2 (TLK1 and 2) are cell cycle-regulated serine/threonine kinases that are involved in multiple biological processes. Mutation of TLK1 and 2 confer neurodegenerative diseases. Recent studies demonstrate that TLK1 and 2 are involved in DNA repair. However, there is no direct evidence that TLK1 and 2 function at DNA damage sites. Here, we show that both TLK1 and TLK2 are hyper-autophosphorylated at their N-termini, at least in part, mediated by their homo- or hetero- dimerization. We found that TLK1 and 2 hyper-autophosphorylation suppresses their recruitment to damaged chromatin. Furthermore, both TLK1 and 2 associate with PCNA specifically through their evolutionarily conserved non-canonical PCNA-interacting protein (PIP) box at the N-terminus, and mutation of the PIP-box abolishes their recruitment to DNA damage sites. Mechanistically, the TLK1 and 2 hyper-autophosphorylation masks the PIP-box and negatively regulates their recruitment to the DNA damage site. Overall, our study dissects the detailed genetic regulation of TLK1 and 2 at damaged chromatin, which provides important insights into their emerging roles in DNA repair.

## Graphical Abstract

**Graphical Abstract:**
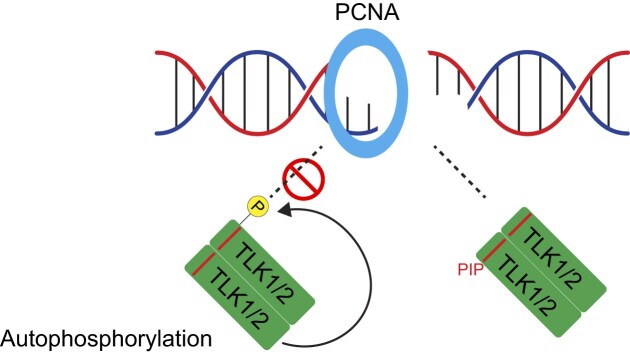

## Introduction

Serine/threonine kinases, Tousled-like kinases 1 and 2 (TLK1 and 2), are evolutionarily conserved among metazoans and were first identified as homologues of the *Arabidopsis* kinase Tousled (1,2). TLK1 and TLK2 are implicated in DNA repair, immune response, chromatin structure, transcription, DNA replication, checkpoint control and cell morphology (2–20)_._ Despite the structural similarity between TLK1 and TLK2, they play a different role in cell survival and development. TLK2, but not TLK1, is required for embryogenesis in mice due to a defect in placental development (9).

Mutations of TLK1 and 2 are associated with neurodevelopmental disorder (11,21–25). Pathological mutations of TLK1 and TLK2 were previously reported. P25L, T38fs and R142S mutations of TLK1 (21) and D551G, E475Ter and S617L mutations of TLK2 are clinically implicated in intellectual disability and developmental defects (11,26). These TLK2 mutations showed reduced kinase activity, increased DNA damage, chromatin compaction defects and mis-localization. Interestingly, a TLK2 mutant with coiled-coil domain 1 deletion showed—perinuclear accumulation (27), coinciding with its reduced dimerization and kinase activity—suggesting that these mutants may also have impaired localization to DNA damage site.

A growing body of evidence shows that TLK1 and 2 are key DNA repair proteins. The activity of TLK is cell-cycle regulated peaking during S-phase progression (14,28) and transiently inhibited after DNA double-strand breaks (DSBs) (29). The DNA damage-induced inhibition is regulated by ATM and Chk1 (29,30). Interestingly, loss of both TLK1 and TLK2 in cells results in increased genomic instability as measured by increased double strand breaks, chromosome bridges and chromosome aberrations that is not present in cells with one functional TLK (9). This suggests that TLK1 and TLK2 serve overlapping roles within the DNA damage response pathway.

Although many TLK substrates have been reported, the H3/H4 histone chaperones, anti-silencing function-1 (ASF1A and ASF1B) (10,31,32), involved in chromatin assembly and DNA repair via CAF1 and HIRA, are the most well characterized (33–38). Besides ASF1A and ASF1B, several DNA repair proteins, including NEK1, RAD9 and RAD54 (18,39–42), were identified as TLK substrates indicating an important role of TLKs in maintaining genome stability (5,42,43). Both TLK1 and TLK2 are enriched at nascent DNA at the replication fork (44). However, with their previously reported functions in stabilizing replication fork and involvement in homologous recombination (3,42), there is no direct evidence that TLKs are localized at the sites of DNA damage.

There are ongoing efforts to develop TLK1 and TLK2 inhibitors for targeting cancer cells with their promising roles in maintaining genome stability (45–47). Emerging evidence demonstrated that TLK1 and TLK2 are involved in cancer progression and therapeutic resistance (48–51) and it has been shown that inhibition of TLK potentiates cell-killing in different types of cancers (3,51–57), making TLK1 and 2 attractive targets for therapeutic strategy development.

Here, we investigated the molecular mechanism by which TLK1 and 2 are regulated at damaged chromatin. Our data suggest that TLK1 and 2 are recruited to laser-induced micro-irradiation and their recruitment is inhibited by their kinase activity. Using proteomic analysis, systematic mutagenesis and biochemical assays, we have identified autophosphorylation sites at the N-terminus of TLK1 and 2. Importantly, auto-hyperphosphorylation of TLK1 and 2 inhibits their recruitment to damaged chromatin. Mechanistically, recruitment of TLK1 and 2 is mediated by PCNA through their putative PCNA interacting protein box (PIP-box) that is masked by the surrounding phosphorylated residues. Our study provides novel molecular insights into TLK activity that regulates their localization to damaged chromatin.

## Materials and methods

### Cell culture

HEK293T and U2OS cell lines were obtained from American Type Culture Collection. U2OS H2AX knockout (KO), RNF168 KO, 53BP1 KO and LC8 KO cells were generated using CRISPR/Cas9 technology as previously described (58). All cells were cultured in Dulbecco’s modified Eagle’s medium with 10% fetal bovine serum supplemented with 100 U/ml penicillin and 100 μg/ml streptomycin at 37°C with 5% CO_2_. Polyethylenimine (PEI) (Polysciences, 24765-1) was used for transfection. PEI and plasmids were mixed in a 3 μl: 1 μg ratio in OPTI-MEM, incubated for 10 min and added to the medium for 6 h.

### Antibodies

Primary antibodies Flag M2 (1:1000, Sigma, F1804), TLK1 (1:1000, Cell Signaling, 4125s), TLK2 (1:1000, Bethyl, A301-257A), Myc (1:1000, Santa Cruz, sc-40), GFP (1:1000, Invitrogen, A11122), β-Tubulin (1:1000, Abcam, ab6046), Histone H4 (1:1000, Cell Signaling, 2935S), LC8 (1:1000, Abcam, ab51603), Thiophosphate ester (1:1000, Abcam, ab92570), Phosphoserine (1:1000, Millipore, AB1603), Phosphothreonine (1:1000, New England Biolabs, 9386S), PCNA (1:1000, Abcam, ab92552), Chk1 (1:1000, Santa Cruz, 8408), Phospho-Chk1 Ser345 (1:1000, Cell Signaling, 2348S), Chk2 (1:1000 Cell Signaling, 3440S), Phospho-Chk2 Thr68 (1:1000, Cell Signaling, 2197S), DNA-PKcs (1:1000, Cell Signaling, 38168S) and Phospho-DNA-PK Ser2056 (1:1000, Invitrogen, PA5-78130) were used for Western blot analysis. Cyclin A (1:1000, BD Biosciences, 611 268) was used for immunofluorescence. Goat anti-mouse IgG-HRP (1:10 000, Jackson ImmunoResearch,111-035-144) and Goat anti-rabbit IgG-HRP (1:10 000, Jackson ImmunoResearch, 115-035-166) secondary antibodies were used for western blotting analysis. Alexa Fluor 594 goat anti-mouse IgG (1:1000, Invitrogen, A11032) was used as a secondary antibody for immunofluorescence.

### Plasmids

Human TLK1 pDONR221 was purchased from the Harvard PlasmID Database (Plasmid #HsCD00042921). Human TLK2 pDONR223 was a gift from William Hahn & David Root (Addgene: Plasmid #23629). Human ASF1A pDONR221 was purchased from the Harvard PlasmID Database (Plasmid #HsCD00376532). Human LC8 expression vectors and its mutants were described previously (58). pDONR vectors were subcloned into indicated gateway-compatible destination vectors using Gateway LR Clonase II (Invitrogen, 11791020). TLK1 and TLK2 point mutants were generated by site-directed mutagenesis reactions using the NEB Q5 site-directed mutagenesis kit (NEB, E0554S). Primers are listed in Supplementary Table S1.

### Tandem affinity purification

Tandem affinity purification (TAP) was carried out as described previously (59). Briefly, S-protein-2xFLAG-Streptavidin binding peptide (SFB)-tagged TLK1, SFB-TLK1 1–240, SFB-TLK1 D607A, SFB-TLK1 133–208, SFB-TLK1 13-208 Y149A F150A or SFB-TLK2 were transfected into HEK293T cells. Cells were harvested 24 h after transfection using NETN buffer (150 mM NaCl, 0.5 mM EDTA, 20 mM Tris–HCl, pH 8.0, 0.5% NP-40) supplemented with 2 μg/ml aprotinin (Thermo, AAJ60237MB) and 5 μg/ml pepstatin A (Thermo, PI78432) at 4°C for 20 min. Lysates were centrifuged at 9000 × g, 4°C for 20 min to yield supernatant as soluble fraction. The pellet was washed 1× with PBS and centrifuged at 9000 × g, 4°C for 5 min and lysed with chromatin extraction buffer (NETN buffer, 10 mM MgCl_2_ and Turbonuclease, Accelagen, N0103M) at 4°C for 1 h followed by centrifugation at 9000 × g, 4°C for 20 min to obtain chromatin fraction. Both soluble and chromatin fractions were incubated with streptavidin sepharose (200 μl) (GE Healthcare, GE17-5113-01) at 4°C for 1 h followed by washing with NETN buffer three times. The protein complexes were eluted with 2 mg/ml biotin at 4°C for 1 h. The eluents were then incubated with S-protein agarose (EMD Millipore, 69704-3) overnight at 4°C, washed three times with NETN buffer, and eluted in 1× Laemmli buffer.

### Mass spectrometric analysis of protein–protein interactions

Protein complexes obtained from TAP were processed by in-gel trypsin digestion and analyzed by tandem mass spectrometry (60). Briefly, protein samples were electrophoresed on a 4–20% tris-glycine gel for 5 min at 300V. The gel was stained with GelCode Safe (Thermo Scientific, 24594) according to the manufacturer’s instructions. The excised gel band was destained three times with 50 mM ammonium bicarbonate (Sigma-Aldrich, A6141) and 50% methanol (Fisher, A456) for 1 h each. Each sample was dehydrated by acetonitrile (Fisher, A955). Gel bands were reduced with 10 mM Tris(2-carboxyethyl)phosphine (TCEP) (Pierce, 20490) for 30 min at 37°C. Free thiol groups were derivatized by 50 mM Iodoacetamide (Sigma-Aldrich, A3221) for 1 h at room temperature. Gel bands were washed by alternating 100 mM ammonium bicarbonate and acetonitrile three times, followed by dehydration with acetonitrile. Samples were digested by sequencing grade trypsin (Promega, V5111) in 50 mM ammonium bicarbonate at 37°C overnight. The samples were then acidified by 0.5% formic acid (Fisher, A117) to a final concentration of 0.1% and were desalted by Water’s C18 SepPak (Waters, WAT023590), dried by SpeedVac and resuspended in 0.1% formic acid. The samples were then injected into an in-line, reverse phase, 150 × 0.075 mm column packed with XSelect CSH C18 2.5 μM resin (Waters, 186006103) using an UltiMate 3000 RSLCnano. Peptides were eluted into an Orbitrap Fusion Tribrid mass spectrometer following a 100-min gradient of 97:3 Buffer A (0.1% formic acid and 0.5% acetonitrile) to buffer B (0.1% formic acid and 99.9% acetonitrile) to 67:33 A:B. Ionization of eluted peptides was performed by electrospray ionization at 2.2 kV. Precursor ions were collected in the Orbitrap at 240 000 resolution for a MS^1^ range of 375 – 1 500 m/z. Data-dependent acquisition was used to select the top 10 precursor ions for fragmentation by higher-energy collisional dissociation with normalized collision energy between 28.0 and 31.0. Fragment ions between 200 and 1400 m/z were measured at MS^2^ in the ion trap.

Proteins were identified using Mascot (Matrix Sciences) to search the *Homo sapiens* UniProtKB database. Search conditions were set to a parent ion tolerance of 3 ppm and a fragment ion tolerance of 0.5 Da. Carbamidomethylation of cysteine was selected for fixed modifications. Oxidation of methionine, acetylation of peptide N-termini and phosphorylation of serine, threonine, and tyrosine were selected as variable modifications. Scaffold (Proteome Software) was used to validate protein and peptide identifications. Proteins and peptides were identified if they exhibited less than 1.0% false discovery by Scaffold’s local false discovery algorithm.

### TMT11plex labeling and phosphopeptide analysis

Peptide labeling and phosphopeptide enrichment were carried out as previously described (61). Briefly, HEK293T cells were transfected with either SFB-TLK1, SFB-TLK2, siRNA targeting TLK1, TLK2, or TLK1 and TLK2. Cells were harvested 24 h after transfection, four replicates of each transfected or control non-transfected cells were lysed in 2% SDS and 100 mM Tris–HCl, pH 7.6 supplemented with fresh protease (Pierce, A32963) and phosphatase inhibitors (Pierce, A32957). Protein concentrations were measured by BCA protein assay (Pierce, 23225). Three hundred microgram of protein from each sample was reduced by TCEP, alkylated by iodoacetamide and purified via chloroform/methanol extraction. Samples were then trypsinized with a protein: trypsin ratio of 50:1 overnight at 37°C followed by quenching with 0.5% formic acid to a final concentration of 0.1%. Samples were salted by Waters C18 SepPaks and eluted peptides were dried using a SpeedVac. One hundred twenty microgram of peptides were labeled using a TMT10plex isobaric label reagent set with the addition of the TMT11-131C label (Thermo, A34808). Labeled peptides were dried by speed-vac and resuspended in phosphopeptides binding/wash buffer from the High-Select TiO_2_ phosphopeptide enrichment kit (Pierce, A32993). Samples were fractionated using a 100 × 1.0 mm Acquity BEH C18 column (Waters) by an Ulitmate 3000 UHPLC system (Thermo) over a 40 min gradient following ratio of 99:1–60:40 basic buffer A:B (buffer B: 10 mM ammonium hydroxide, 99.9% acetonitrile, pH 10) into 36 fractions. Multinotch MS^3^ was performed as previously described (62). Data analysis was performed using Maxquant (Max Planck Institute) (61,63). The *Homo sapien* UniprotKB database was used for peptide searching, intensities were used to determine significantly enriched phosphopeptides by the ProteoViz R scripts with the R package limma (64,65). Heatmaps were created in GraphPad Prism 10 (GraphPad). Pathway analysis by EnrichR was performed on proteins with enriched phosphosites, cutoff was set at log_2_ fold change >1 with adjusted *P*-value <0.05 (66).

### Streptavidin pulldown

HEK293T cells were transfected with SFB-expression vectors as indicated and harvested by addition of trypsin, washed with PBS, and then lysed by addition of NETN buffer with Turbonuclease for 1 h at 4°C. Lysates were centrifuged at 14 000 × g, 4°C for 20 min. Supernatants were incubated with streptavidin sepharose beads for 1 h at 4°C with rotation and washed three times with NETN buffer. Protein complexes were eluted 1× Laemmli buffer for Western blotting analysis.

### Chromatin fractionation

HEK293T cells were transfected with the SFB-expressing vectors. Cells were harvested NETN buffer supplemented with aprotinin and pepstatin A. Whole cell extracts were obtained from the direct lysate. The remaining lysate was centrifuged at 14 000 × g, 4°C for 20 min. The supernatants were collected for the soluble fraction and the insoluble chromatin pellet was washed twice with PBS. The pellets were extracted using 0.2N hydrochloric acid for 15 min as the chromatin fraction and neutralized by equal volume 1M Tris, pH 8.0.

### Kinase inhibitor treatment

U2OS cells with TLK transfection were treated with either DMSO, 10 μM ATM inhibitor (KU55933, Selleck Chemicals, S1092), 10 μM ATR inhibitor (VE-821, Selleck Chemicals, S8007) or 2 μM DNA-PK inhibitor (NU7441, Selleck Chemicals, S2638) for 1 h as indicated followed by western blotting analysis and laser-induced micro-irradiation experiment.

### Western blotting

Samples were resolved by SDS-PAGE, transferred to PVDF membranes, blocked with 5% non-fat milk or 3% BSA and incubated with the indicated primary antibodies overnight at 4°C. The blots were washed for three times with 1× Tris-Buffered Saline with 0.1% Tween 20 (TBST). The membranes were incubated at room temperature for 1 h with HRP-conjugated secondary antibodies. The blots were then washed three times with TBST and were developed using Clarity ECL (BioRad, Cat #1705061). Images were captured using a BioRad ChemiDoc MP.

### siRNA-mediated knockdown

SiRNA-mediated knockdown of PCNA in U2OS cells was performed using Lipofectamine RNAiMAX (Invitrogen, 13778075) following manufacturer’s instructions. 60 pmol of PCNA MISSION esiRNA (Sigma-Aldrich, EHU105721-20UG) was transfected for 4 h each time in two consecutive days followed by GFP-TLK1 D607A or GFP-TLK2 D613A transfection. Knockdown efficiency was confirmed using specific antibody by western blotting analysis.

### Laser-induced micro-irradiation

Cells were seeded on 35 mm glass bottom dishes (Cellvis, D35-20-1.5-N) and transfected with GFP-tagged proteins as indicated. Laser-induced micro-irradiation was performed using a Nikon Ti2 inverted fluorescent microscope and C2+ confocal system with an Okolab live cell imaging chamber attached to a gas-mixer maintained at a temperature of 37°C and 5% CO_2_. The 405 nm laser of the C2+ confocal system was used to irradiate each cell at the indicated region at 100% laser power for 2 s. Images were captured in 30 s intervals after damage and the fluorescent intensity of GFP-tagged protein recruitment to the damaged region was quantified. For cyclin A staining, cells were subjected to laser-induced micro-irradiation and harvested after 10 min followed by immunofluorescence staining with indicated antibodies. Laser-induced micro-irradiation experiments for GFP-TLK1 D607A or GFP-TLK2 D613A in PCNA knockdown cells was performed using a ZEISS confocal laser scanning microscope LSM900 with comparable setting as the C2+ confocal in the same study. The intensity of the recruitment was normalized by dividing the background intensity of an undamaged area within the same cell at the same time point.

### Kinase assays

Kinase assays were performed as previously described (67). Briefly, SFB-tagged proteins were expressed in HEK293T cells for 48 h. Cells were lysed in NETN buffer with protease inhibitors, 10 mM MgCl_2_ and Turbonuclease for 1 h at 4°C. Samples were centrifuged at 14 000 × g, 4°C for 20 min. Supernatants were incubated with S-protein beads for 1 h at 4°C. Beads were washed three times with NETN, twice with kinase wash buffer (40 mM HEPES, pH 7.5, 250 mM NaCl), and once with kinase reaction buffer (30 mM HEPES, pH 7.5, 50 mM potassium acetate, 5 mM MgCl_2_). Equal volumes of SFB-tagged proteins in kinase reaction buffer and 500 μM ATP-γ-S (Abcam, ab138911) were mixed and incubated at 30°C for 30 min. ATP- γ-S was alkylated by p-Nitrobenzyl mesylate (PNBM, Abcam, ab138910) to a final concentration of 2.5 mM and incubated at room temperature for 1 h. Alkylation was terminated using 2× Laemmli buffer. Phosphorylation was detected after Western blotting by an anti-thiophosphate ester antibody.

### Lambda protein phosphatase assay

HEK293T cells were transfected with SFB-expressing vectors. SFB-tagged proteins were purified using S-protein beads and washed for three times with NETN buffer. SFB-tagged proteins were aliquoted into a fresh 1.5 ml tube containing 1× NEBuffer for Protein MetalloPhosphatases supplemented with 1 mM MnCl_2_, and 400 units of Lambda protein phosphatase (λpp) (New England Biolabs, P0753S). The reactions were incubated at 30°C for 30 min and terminated by an equal volume of 2× Laemmli buffer.

### Statistical analysis

Laser-induced micro-irradiation experiments represent the mean ± SEM of *N* ≥ 10 cells in each condition unless otherwise specified. Unpaired, two-tailed Student’s *t-*tests were performed in GraphPad Prism 10. Statistical significance corresponds to *P* < 0.05.

## Results

### TLK1 and TLK2 form homo- and heterodimers

Recent studies revealed that LC8 (also known as DYNLL1) is a DNA repair protein that functions through its interactions with 53BP1, a key regulator of DSB pathway choice (58,68–70). Previously, we demonstrated the binding of LC8 to numerous DNA repair proteins, including CHD4, ZMYM2 and ZMYM3. Consistent with previous reports (27), we observed TLK1 and TLK2 were pulled down as LC8 interactors in our LC8 proteomic analysis (9,58). Subsequent TAP-coupled mass spectrometry of TLK1 and TLK2 revealed that TLK1 and TLK2 are each other’s primary interactors followed by LC8 (Supplementary Figure S1A).

**Figure 1.**
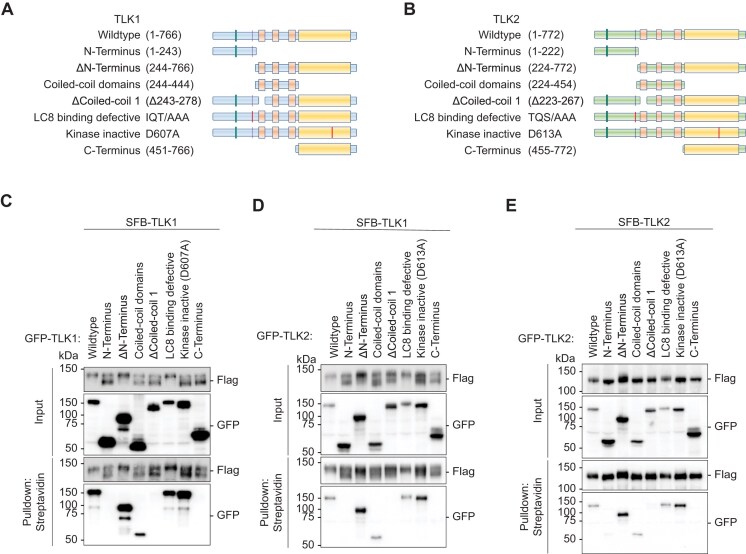
Tousled-like kinases form dimers and drive autophosphorylation. (**A**and**B**) Schematic illustrations of TLK1 and TLK2 constructs for dimerization domain mapping. (**C**–**E**) TLK dimerization requires an intact coiled-coil domain. HEK293T cells were co-transfected with (**C**) SFB-TLK1 wildtype and the indicated GFP-TLK1 expression vectors, (**D**) SFB-TLK1 wildtype and GFP-TLK2 plasmids, (**E**) SFB-TLK2 and GFP-TLK2 plasmids, and pulldown experiment followed by Western blotting analysis using FLAG and GFP antibodies.

In order to map the interactions between TLK1/2 and LC8, we systematically mutated the known domains of TLK1 and 2 (Figure 1A and B) and performed pulldown assays (Figure 1C–E). Consistent with previous work, we found that TLK1 and TLK2, both form homodimers and heterodimers through their coiled-coil 1 domain (Figure 1C–E) (27,71). We identified the LC8 anchoring motifs on TLK1 (SFKI**IQT**D) and TLK2 (QHEQ**TQS**D) (Supplementary Figure S1B–H) as direct interaction sites for the LC8 canonical binding groove. However, LC8 was not involved in TLK1 and TLK2 dimerization (Figure 1C–E) despite the well-characterized function of LC8 in promoting the dimerization of its interacting partners, including other kinases such as NEK9 (58,69,72).

### Dimerization of TLK1 and TLK2 drives autophosphorylation of the N-terminus

**Figure 2.**
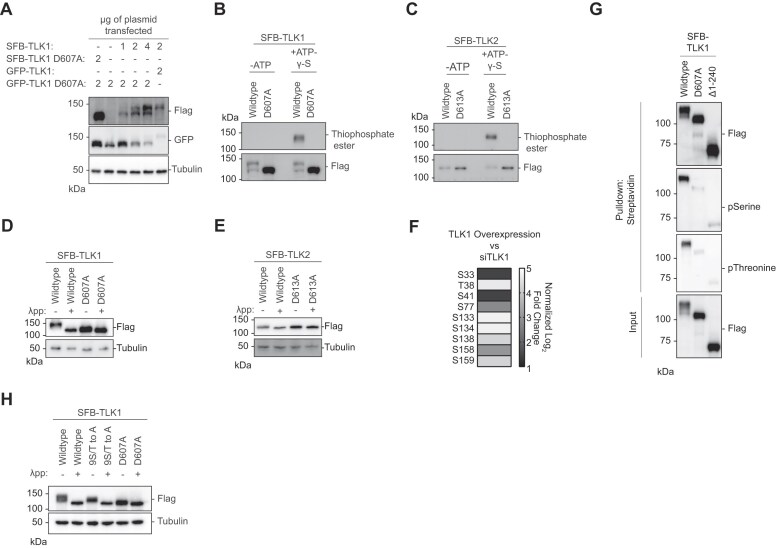
Tousled-like kinases are autophosphorylated at their N-terminus. (**A**) Co-transfection of TLK1 wildtype and catalytic inactive mutant D607A with the indicated amount of plasmids in HEK293T cells followed by Western blotting analysis. (**B**and**C**) Kinase assays using ATP-γ-S followed by alkylation by p-Nitrobenzyl mesylate to convert phosphorylated residues into thiophosphate esters. Antibody against thiophosphate ester was used to identify phosphorylation events. (**D**and**E**) Lambda protein phosphatase (λpp) assay was performed using SFB-tagged TLK1 wildtype and D607A mutant or SFB-tagged TLK2 wildtype and D613A mutant purified from HEK293T cell lysate. Differential molecular weights were analyzed by Western blotting analysis. (**F**) TLK1 is autophosphorylated at nine residues within its N-terminus. Phospho-Tandem-Mass-Tag mass spectrometry was performed on phosphopeptides enriched from HEK293T cells with TLK1 overexpression and knockdown using siRNA. (**G**) TLK1 N-terminus is highly phosphorylated. SFB-tagged TLK1 wildtype, D607A or Δ1–240 (ΔN-terminus) were purified from HEK293T using streptavidin beads followed by Western blotting analysis using phospho-Serine and phospho-threonine antibodies. (**H**) TLK1 wildtype, N-terminal 9S/T to A and catalytic inactive mutants were treated with λ-phosphatase. Differential molecular weights were analyzed by Western blot.

In the pulldown assay, we observed a pronounced differential migration rate for TLK1, and a modest difference for TLK2, when co-expressed with different mutants (Figure 1C–E). More specifically, SFB-TLK1 migrated slower when co-expressed with wildtype, ΔN-terminus, and LC8-binding defective mutants. Co-expression with the non-interacting (N-terminus, coiled-coil domain, Δcoiled-coil1 and C-terminus) or the catalytically inactive D607A mutants revealed two prominent forms: a faster and slower migrating TLK1 (Figure 1C and D). Interestingly, co-expression of GFP-TLK1 and 2 wildtype, ΔN-terminus and LC8-binding defective mutants shifted the SFB-TLK1 D607A to the slower migrating form (Supplementary Figure S2A and B), while we only observed the faster migrating bottom band for SFB-TLK1 D607A upon co-expression with non-interacting and catalytic inactive mutants (Supplementary Figure S2A and B). Consistently, a TLK2 catalytically inactive mutant did not show a drastic shift in size when co-expressed with wildtype or a number of different mutants (Supplementary Figure S2C). The mobility shift was dependent on the presence of the active form of TLK1. We only observed a faster migrating band when we co-expressed SFB-TLK1 D607A and GFP-TLK1 D607A (Figure 2A). Titrating the wildtype plasmid with GFP-TLK1 D607 mutant construct, we observed a mobility shift to a slower migrating band (Figure 2A). The mobility shift correlated with the presence of TLK1 activity, indicating that the upper band of TLK1 is the phosphorylated form.

Consistently, kinase assays showed that wildtype TLK1 had slower electrophoretic mobility compared to the catalytic inactive TLK1 D607A mutant (Figure 2B), which showed no detectable activity (Figure 2B and C). To confirm that the increased mobility was due to phosphorylation, wildtype TLK1 was treated with lambda phosphatase. Treated TLK1 showed faster electrophoretic mobility similar to corresponding catalytically inactive mutants (Figure 2D), consistent with our previous observations (Figure 1C and D). In contrast, the difference in migration between wildtype TLK2 and the catalytically inactive mutant was less pronounced (Figure 2C–E), which may correlate with reduced phosphorylation levels. Using quantitative phospho-enrichment proteomics, we identified nine residues at the TLK1 N-terminus that were phosphorylated (Figure 2F). Additionally, we identified a list of potential TLK1 substrates that are involved in DNA repair as classified by Gene Ontologies (Supplementary Table S2). Using phospho-serine and phospho-threonine antibodies, we confirmed that the TLK1 N-terminus is the primary target for auto-phosphorylation (Figure 2G). Mutation of the nine serine/threonine residues to alanine (9S/T to A) at the N-terminus increased the electrophoretic mobility (Figure 2H) indicating that mutation of 9S/T to A reduces, but does not abolish autophosphorylation. Together, these data suggest that active TLK1 and TLK2 are autophosphorylated and this is mediated in trans through homo- or heterodimerization.

### TLK1 and 2 kinase activity regulates their localization at DNA break sites

**Figure 3.**
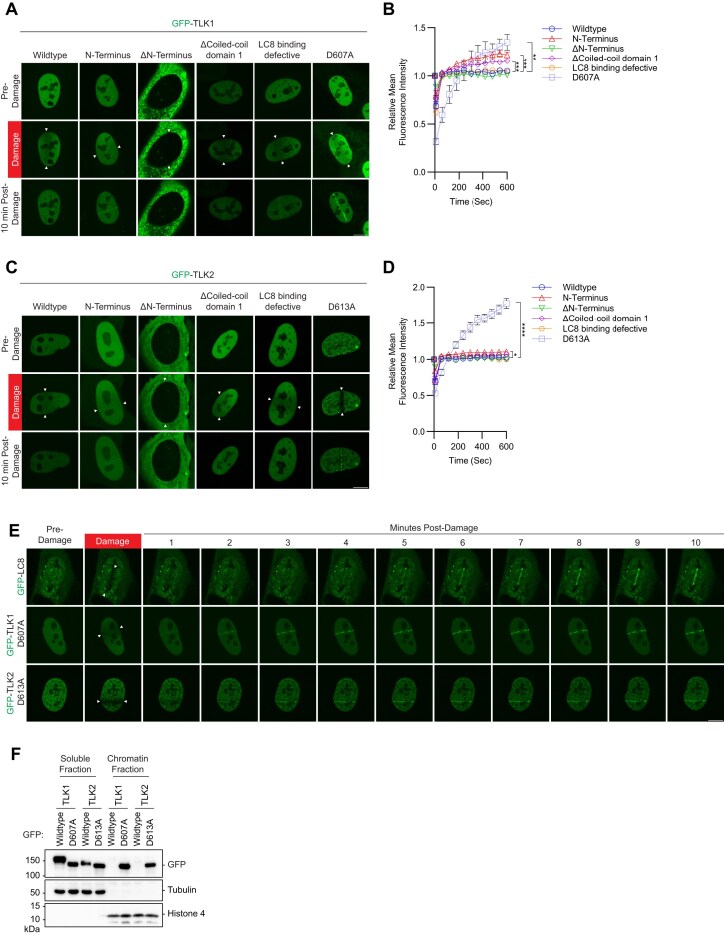
Accumulation of TLK1 and 2 is negatively regulated by their kinase activity and dimerization at damaged chromatin. (**A**and**C**) TLK1 and TLK2 recruitment to sites of DNA damage is repressed by dimerization and kinase activity. U2OS cells were transfected with indicated GFP-TLK1 or TLK2 wildtype and mutants. Laser-induced micro-irradiation and live-cell imaging experiments were conducted using a confocal microscope, denoted line between the white arrows. Representative images of each experimental group at 10 min after damage. Scale bar represents 10 μm. (**B, D**) Quantification of laser-included micro-irradiation as in (A) and (C), respectively. *N* ≥ 10 cells. Significance was determined by unpaired, two-tailed Student’s *t*-test and *P*-values are reported as *P < 0.05, **P < 0.01, ***P < 0.001 and ****P < 0.0001. (**E**) DNA damage kinetics of LC8, TLK1 and TLK2 catalytic inactive mutants using laser-induced micro-irradiation. Scale bar represents 10 μm. (**F**) Kinase activity negatively regulates TLK chromatin loading. HEK293T cells were transfected with the indicated GFP-TLK1 wildtype and catalytic inactive mutants. Cells were harvested 24 h after transfection and fractionated to obtain soluble and chromatin fractions.

LC8, a recently identified DNA repair factor, is recruited to DNA breaks (58). Although LC8 interacts with both TLK1 and TLK2, we did not observe GFP-TLK1 or TLK2 accumulation at DNA damage sites induced by laser micro-irradiation (Figure 3A–D). Surprisingly, in both TLK1 and TLK2, the N-terminus, coiled-coil 1 domain deletion, and catalytically inactive mutants robustly accumulated at sites of DNA damage (Figure 3A–D). In contrast, the N-terminus deletion mutants were primarily cytoplasmic and did not localize to sites of damage (Figure 3A–D). The catalytically inactive mutants GFP-TLK1(D607A) and TLK2 (D613A) localized at DNA damage sites within 3 min, with faster kinetics compared to LC8, which localizes to laser-induced damaged DNA at 4–5 min (Figure 3E). Moreover, LC8 did not appear to play a role in regulating TLK1 and TLK2 recruitment to DNA damage as its depletion did not alter their localization kinetics (Supplementary Figure S3A–H). Wildtype TLK1 is not recruited to laser-induced micro-irradiation even for a 1 h time course experiment, while the TLK1 D607A retained at DNA damage site for at least 1 h (Supplementary Figure S4A and B). And the recruitment of TLK1 D607A is not cell-cycle dependent (Supplemental Figure S4C and D). Consistently, fractionation experiments showed that catalytically dead mutants of TLK1 and TLK2, but not their wildtype counterparts, were highly enriched in the chromatin fraction (Figure 3F). Together, our data suggest that the accumulation of TLK1 and 2 at damaged chromatin is primarily mediated by their N-terminus and inhibited by autophosphorylation that occurs independently of LC8.

### N-terminal autophosphorylation inhibits TLK recruitment to DNA damage sites

**Figure 4.**
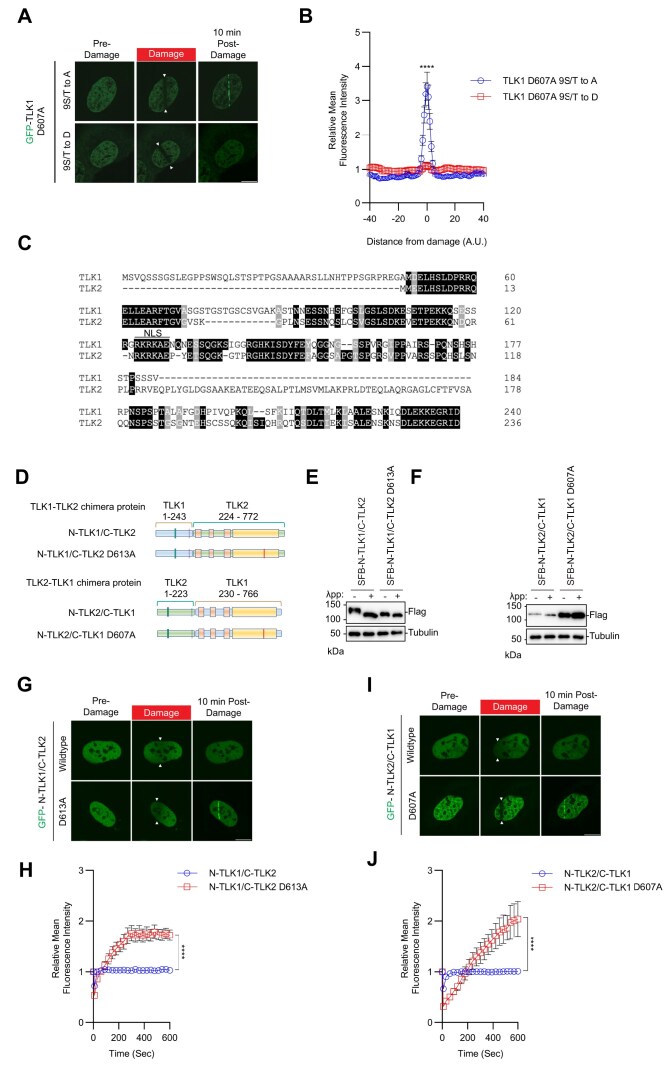
Recruitment of TLK1 and 2 to damaged DNA is N-terminal phosphorylation-dependent. (**A**) TLK1 autophosphorylation suppresses recruitment to damaged chromatin. The TLK1catalytic mutant (D607A) with N-terminus phospho-defective mutant (9S/T to A) or phospho-mimetic mutant (9S/T to D) were subjected to laser-induced micro-irradiation. Scale bar represents 10 μm. (**B**) Quantification of the recruitment intensity as shown in A. *N* = 10. (**C**) Sequence alignment of the N-terminus of TLK1 and TLK2. (**D**) Schematic illustration of chimeric proteins generated for TLK1 and TLK2. (**E**and**F**) Chimeric N-TLK1/C-TLK2, N-TLK1/C-TLK2 D613A, N-TLK2/C-TLK1 and N-TLK2/C-TLK1D607A were treated with λpp. Apparent molecular weights were analyzed by Western blot. (**G**) Representative images of GFP- N-TLK1/C-TLK2 and N-TLK1/C-TLK2 D613A chimeras at 10 min after laser-induced micro-irradiation. The damaged area was denoted between the arrows. Scale bar represents 10 μm. (**H**) Recruitment kinetics quantification of the GFP-chimera proteins after laser-induced micro-irradiation as in (**G**). *N* ≥ 10. (**I**) Representative images of GFP- N-TLK2/C-TLK1 and N-TLK2/C-TLK1 D607A chimeras at 10 min after laser-induced micro-irradiation. The damaged area was denoted between the arrows. Scale bar represents 10 μm. (**J**) Recruitment kinetics quantification of the GFP-chimera proteins after laser-induced micro-irradiation as in (**I**). *N* ≥ 10. Significance was determined by unpaired, two-tailed Student’s *t*-test and *P*-values are reported as *P < 0.05, **P< 0.01, ***P< 0.001 and ****P < 0.0001.

Given the robust recruitment of the N-terminal fragment and the catalytically inactive mutants of TLK1/2 to laser-induced micro-irradiation, we tested whether the recruitment was dependent on the phosphorylation of the N-terminus. Strikingly, the phosphomimetic mutant (9S/T to D) abolished GFP-TLK1 D607A recruitment to DNA damage sites, whereas the 9S/T to A mutant retained robust recruitment of GFP-TLK1 D607A to DNA damage (Figure 4A and B). This suggested that the TLK1 N-terminal auto-phosphorylation sites may be involved in suppressing its retention at DNA damage sites and the regulation is not sequence-specific because of the 9S/T to A D607A mutant can still be recruited to damaged chromatin.

We observed a discernible mobility shift in TLK1 9S/T to A mutant with Lambda protein phosphatase treated groups suggesting the N-terminal 9S/T are not the only phosphorylated residues. Treatment with inhibitors to the PI3K-related kinases (ATM, ATR and DNA-PKcs) did not further reduce the TLK1 phosphorylation state, while TLK1 catalytic mutant is comparable to the Lambda protein phosphatase treatment suggesting that the TLK1 autophosphorylation activity may also target other sites (Supplementary Figure S5A and B). Interestingly, inhibition of ATR reduced accumulation of both TLK1 and TLK2 at sites of DNA damage, while ATM and DNA-PKcs inhibition showed modest or insignificant reduction of TLK1 and TLK2 recruitment (Supplementary Figure S5C–F). These data suggest that ATR may play an indirect role in regulating TLK1 and TLK2 accumulation.

To confirm that the robust mobility shift of the wildtype TLK1 is due to the hyperphosphorylation of its N-terminus, a region where TLK1 and TLK2 share low sequence homology (Figure 4C), we performed a domain-swapping experiment by fusing the N-terminus of TLK1 with the TLK2 coiled-coil domains and kinase domain and vice versa (Figure 4D). Chimeric protein consisting of TLK1 N-terminus-TLK2 C-terminus (N-TLK1/C-TLK2) showed slower electrophoretic mobility compared to the TLK1 N-terminus-TLK2 C-terminus catalytically inactive (N-TLK1-C-TLK2D613A) chimeric protein, indicating the shift was due to autophosphorylation. Consistent with the lower mobility of wildtype TLK2 compared to TLK1, the N-TLK2/C-TLK1 chimera showed a small mobility difference compared to the N-TLK2/C-TLK1 D607A. Although both TLK1 and TLK2 are auto-phosphorylated at their N-termini, the TLK1 N-terminal hyperphosphorylation results in a more pronounced mobility shift (Figure 4E and F). Despite this difference, their N-terminal autophosphorylation consistently regulated their recruitment to DNA damage (Figure 4G–J).

**Figure 5.**
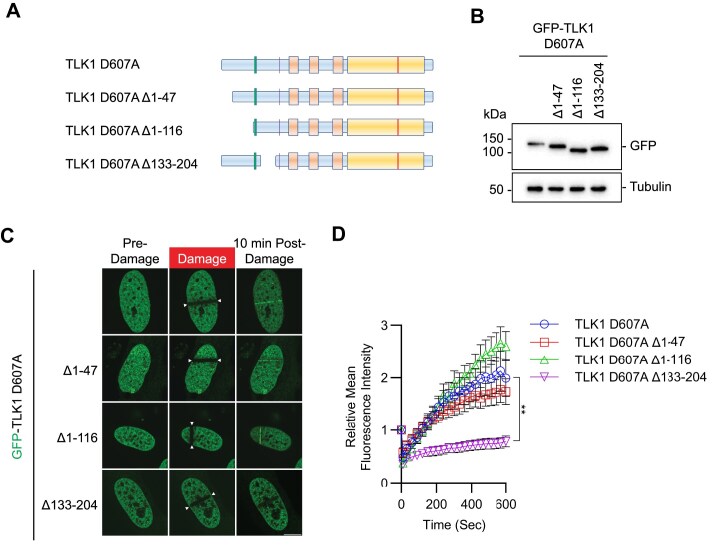
The N-terminus conserved region of TLK1 is required for its laser-induced micro-irradiation. (**A**) Schematic illustrations of TLK1 N-terminal deletion mutants for damaged chromatin recruitment region mapping. (**B**) Western blotting analysis of the mutants as in A and used in C and D. (**C**) Representative images of TLK1 N-terminal deletion mutants 10 min after laser-induced micro-irradiation. Scale bar represents 10 μm. (**D**) Recruitment kinetics of the TLK1 N-terminal deletion mutants as in C. *N* ≥ 10. Significance was determined by unpaired, two-tailed Student’s *t*-test and *P*-values are reported as *P < 0.05, **P <0.01, ***P < 0.001 and ****P < 0.0001.

To pinpoint the N-terminal domain of TLK1 required for the recruitment to DNA damage sites, we generated systematic serial deletions of TLK1 at the N-terminus, without perturbing the nuclear localization signal, on the wildtype, D607A, and N-terminus (1–240) backbone. Deletion of the first 47 amino acids (Δ47), or first 116 (Δ116), did not show significant change in TLK1 D607A recruitment to DNA damage (Figure 5A–D, Figure 4C). Strikingly, deletion of 133–204 (Δ133–204), where five hyperphosphorylated residues (S133, S134, S138, S158 and S159) reside, completely abolished TLK1 D607A recruitment to DNA damage (Figure 5C and D). Consistently, the N-terminus (1–240) Δ133–204 mutant showed a dramatic reduction of recruitment to DNA damage when compared to the wildtype 1–240 fragment (Supplementary Figure S6A–D). As expected, the deletion did not alter the DNA damage recruitment of wildtype TLK1 due to the hyperphosphorylation of TLK1 at the basal level (Supplementary Figure S6E–H).

### PCNA recruits TLK1 and TLK2 DNA damage sites via protein–protein interaction

**Figure 6.**
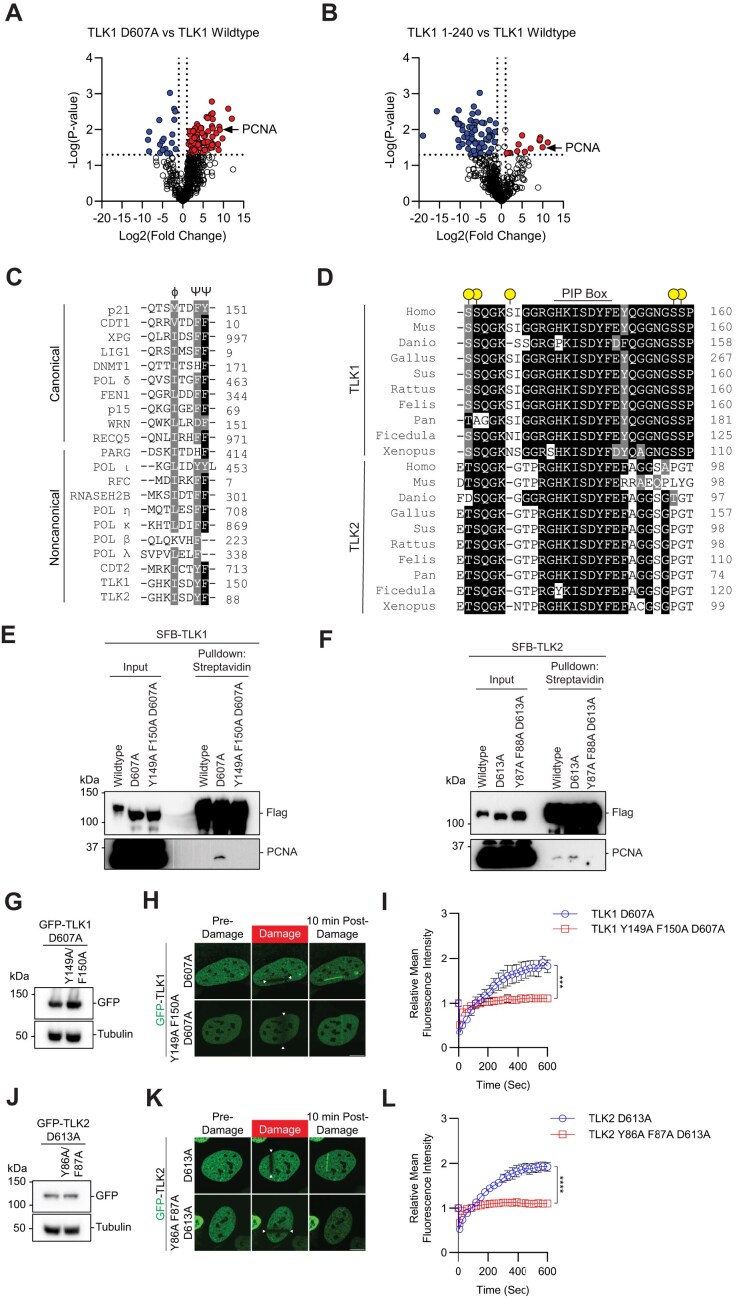
PCNA regulates TLK1 and TLK2 DNA damage recruitment via interaction with their PIP-box. (**A**and**B**) Volcano plot of proteomic analyses between TLK1 wildtype and catalytic mutant D607A or N-terminal fragment a.a. 1–240. Data was analyzed using Perseus and represents technical duplicate data. (**C**) Sequence alignment of the canonical and non-canonical PIP-box between TLK, TLK2 and other PCNA interacting proteins. PIP-box QxxϕxxΨΨ, where x represents any amino acid, ϕ represents hydrophobic residues and Ψ represents aromatic residues) or non-canonical sequences (xxxϕxxΨΨ). (**D**) Sequence alignment of the TLK1 and TLK2 PIP-box across species. Dot labels represent identified TLK1 autophosphorylation sites. (**E**and**F**) TLK1 and TLK2 interact with PCNA. HEK293T cells transfected with SFB-TLK1 wildtype, D607A or PIP box mutant Y149A F150A D607A. Cells were pulled down by streptavidin beads and analyzed by western blot using indicated antibodies. (G–I) TLK1 PIP box is required for DNA damage recruitment. (**G**) GFP-TLK1D607A and D607A PCNA binding defective mutant (Y149A/F150A) protein expression. (**H**) Representative images of indicated mutants at 10 min after laser-induced micro-irradiation. The irradiated area is induced by the arrows. (**I**) DNA damage recruitment kinetics quantification as in (H). *N* = 10. Scale bar represents 10 μm. (J–L) TLK2 PIP box is required for DNA damage recruitment. (**J**) GFP-TLK2D613A and D613A PCNA binding defective mutant (Y86A/F87A) protein expression. (**K**) Representative images of indicated mutants at 10 min after laser-induced micro-irradiation. The irradiated area is induced by the arrows. (**L**) DNA damage recruitment kinetics quantification as in (K). *N* ≥ 10. Significance was determined by unpaired, two-tailed Student’s *t*-test and *P*-values are reported as *P < 0.05, **P < 0.01, ***P < 0.001 and ****P < 0.0001. Scale bar represents 10 μm.

To dissect the mechanism by which TLK1 and TLK2 are recruited to damaged chromatin, we used a comparative proteomic approach to identify the upstream mediator for the recruitment of TLK to damaged chromatin. We performed TAP-MS using TLK1 wildtype, D607A mutant, and 1–240 fragments as baits. Consistent with previously reported TLK-PCNA interaction identified by proteomic screens (73), we found that PCNA was enriched in both the TLK1 D607A and 1–240 fragments (Figure 6A and B), both of which were recruited to DNA damage induced by laser-induced micro-irradiation (Figure 3A). Within the region between a.a. 133–240, sequence analysis showed that both TLK1 and TLK2 contain a non-canonical PCNA interacting protein-box (PIP box) ϕxxΨΨ (74,75) (Figure 6C), which is highly evolutionarily conserved (Figure 6D). Pulldown assays showed that both the TLK1 and TLK2 catalytically inactive mutants had increased binding affinity to PCNA compared to their wildtype counterpart and mutation of the PIP-box abolished the PCNA binding to the catalytically inactive mutants. TLK2 wildtype showed a low level of PCNA binding, potentially due to its low basal level of hyperphosphorylation at the N-terminus (Figure 6E and F). Furthermore, we were able to detect PCNA with mass spectrometry using a small TLK1 fragment (a.a. 133–208), but not the PIP-box mutant, indicating their specific interaction (Supplementary Figure S7A). Notably, the PIP-box mutation abolished the recruitment of TLK1 and TLK2 catalytically inactive mutants to laser-induced micro-irradiation DNA damage (Figure 6G–L), as well as in the small fragment (Supplementary Figure S7B and C). Consistently, PCNA depletion abolished TLK1 D607A and TLK2 D613A recruitment to laser-induced micro-irradiation (Supplementary Figure S7D–H). Mutation of TLK1 N-terminal autophosphorylation sites to alanine modestly increased the interaction of PCNA compared to catalytically active TLK1 but still significantly weaker compared to the D607 catalytic mutant (Supplementary Figure S8A). This observation suggests that the phosphorylation status of TLK1’s N-terminal nine serine and threonine residues may regulate PCNA interaction. The TLK1 PIP-box mutation did not affect its interaction with other protein, such as its well-characterized substrate ASF1A. (Supplementary Figure S8B). Together, our data reveal that N-terminal autophosphorylation of TLK1 and TLK2 inhibited the interaction with PCNA and regulated their recruitment to DNA damage.

## Discussion

Emerging evidence demonstrates that TLK1 and TLK2 are involved in a wide range of cellular processes, including development, DNA replication, cell cycle regulation and transcription (5,76). Pathophysiologically, the inactivation of TLK1 and TLK2 leads to DNA damage, innate immune activation, induction of alternative lengthening of telomeres pathway and increased replication stress (3,8). The functions of TLK1 and TLK2 have been widely investigated through their interactors and substrates, including ASF1A, ASF1B, NEK1, RAD9, DYNLL1/LC8 and RAD54 (6,10,27,31,33,42,76). Yet, the accumulation of TLK1 and TLK2 at DNA damage sites and the mechanisms underlying their chromatin recruitment have never been shown.

In this study, we found that TLK1 and TLK2 accumulate at DNA damage sites, specifically in their inactive state. Their DNA damage recruitment is mediated by interactions with PCNA that are negatively regulated by their dimerization-mediated N-terminal auto-phosphorylation. We identified nine major sites at the N-terminus that are subjected to autophosphorylation. The residual phosphorylation events are also mediated by autophosphorylation as kinase inactive mutant demonstrated a stronger reduction in phosphorylation than either the 9 S/T to A mutant alone or with PI3K-related kinase inhibitors (Supplementary Figure S5B). Nevertheless, the functions of these robust autophosphorylations await characterization. TLK1/2 activity is inactivated after multiple types of DNA damage, including IR, UV irradiation and hydroxyurea treatment (29). We speculate that the activity-dependent interaction mechanism rapidly relocalizes TLK1 and TLK2 to sites of damage and allows them to phosphorylate substrates at the break sites and dynamically dissociate from chromatin. It is also possible that TLK1 and TLK2 have biological functions at damaged chromatin that are independent of their activity. Additionally, TLK1/2 activity is cell cycle-dependent with the highest activity in early S-phase and declining over mid and late S-phase (29), highlighting its role in targeting the ASF1 histone chaperone throughout replication that may not occur on chromatin.

Overexpression of the truncated TLK1 isoform, TLK1B, lacking the first 237 amino acids has been shown to cause radioresistance in mouse fibroblast cells (4). This isoform lacks the PIP-box found in full-length TLK1 which potentially mediates the upstream regulation of TLKs. However, ectopic expression of TLK1B may be able to bypass this regulatory mechanism. We believe that the endogenous TLK1B may not function at the sites of DNA damage.

TLK1, but not TLK2, has been shown to phosphorylate RAD9 *in vitro*, a component of the 9-1-1 (RAD9-HUS1-RAD1) complex (40,41). Ectopic TLK1B expression leads to RAD9 phosphorylation resulting in dissociation of RAD9 from stalled replication forks. The dysregulation of the 9-1-1 complex hyperactivates S-phase checkpoint and delays recovery (18). TLK1 overexpression appears to be always active regardless of the PCNA binding, it is challenging to precisely dissect its regulation using a depletion-reconstitution system. We believe that the endogenous TLK1 localization via PCNA and RAD9 targeting is a multi-layer regulatory mechanism to ensure replication fork stability, and proper checkpoint control. Hyperactivation or inactivation of TLK leads to checkpoint dysregulation (3,18). Since TLK1 and TLK2 are functionally distinctive, yet share redundancy, their precise roles in maintaining genome stability could be context specific. Detailed investigation using genetic and pharmacological approach will provide better understanding into the roles of TLKs.

Our data showed that the autophosphorylation of residues surrounding TLK1’s PIP-box, specifically S133, S134, S138, S158 and S159, inhibits TLK1’s interaction with PCNA (Figure 6E, F and Supplementary Figure S8A). This could potentially be through the addition of phosphate groups creating steric hinderance to the protein–protein interface.

During homologous recombination, PCNA is loaded on the strand invaded D-loop and we speculate that this could result in the recruitment of TLK1 and TLK2 there for the regulation of repair factors at damaged chromatin, including RAD54 and ASF1 (37,42,77,78). In line with a recent study, the hypersensitivity of TLK depletion implicates their potential involvement in homologous recombination repair (3).

Besides the PCNA binding at the TLK1 and TLK2 N-terminus, LC8/DYNLL1 shows constitutive binding through the putative LC8 binding motif on TLK1 (SFKI**IQT**D) and TLK2 (QHRQ**TQS**D). In contrast to the 53BP1-LC8 interaction, there is no apparent regulatory role for LC8 binding to TLK1 or TLK2 in the context of their recruitment to damaged chromatin. Moreover, despite the well-characterized role of LC8 in regulating protein dimerization (72), LC8 is not required for TLK1 and TLK2 dimerization. The functional connection between LC8 and TLKs thus awaits future investigation.

TLK1 and TLK2 appear to constitutively form higher-order oligomers (27). However, it is unclear how they stoichiometrically dimerize or oligomerize in physiological and pathological conditions. As we observed that dimerization is essential for their autophosphorylation, we speculate that at least the initial activation and phosphorylation occurs in trans and the constitutive autophosphorylation is maintained by both in trans and in *cis* activity. TLK1 and TLK2 coiled-coil 1 deletion are capable of recruitment to sites of DNA damage. There is an increased wildtype mobility when co-transfecting with the TLK1 coiled-coil deletion mutant (Figure 1C and D), suggesting that TLK1 phosphorylation is at least in part contributed by dimerization-mediated in trans autophosphorylation. We noted that recruitment of TLK coiled-coil 1 deletion is significantly lower than that of the catalytic mutants. Although the N-terminus alone, which contains the PIP-box without either in *cis* or in trans phosphorylation, is recruited to laser-induced micro-irradiation, the intensity of recruitment is low. We believe that an unidentified mechanism promotes TLK recruitment to DNA damage site, which could be mediated by the coiled-coil domain.

The diverse biological roles of the DDR pathway suggest that dysregulation of TLK localization at damaged chromatin could significantly impact replication, homologous recombination repair, and histone deposition. Both TLK1 and TLK2 are recruited to sites of DNA damage via interactions with PCNA, mediated by their autophosphorylation. However, dissecting their specific biological functions remains challenging due to the overlap and redundancy in their roles, which are not yet fully characterized. Proper repair function relies on the tight regulation of TLK protein levels and activities within the cell, as highlighted by the transient inactivation of TLKs following DNA damage. Notably, overexpression of TLK1 has been associated with poor prognosis in various cancers and increased genomic instability (15,76). Our data indicate that TLK overexpression results in hyper-autophosphorylation, impairing recruitment to DNA damage sites and compromising repair fidelity. Conversely, the absence of autophosphorylation-mediated regulation prevents context-specific interactions between TLKs and PCNA. Given the intricate regulation of TLKs, future studies using gene-editing approaches to introduce specific mutations in both the catalytic residues and PIP boxes of TLK1 and TLK2 are essential. These investigations will clarify the role of PCNA-mediated TLK recruitment to DNA damage and the biological significance of the autophosphorylation mechanism.

Overall, our study provides strong evidence that TLK1 and 2 accumulate at DNA damage sites via autophosphorylation-regulated PCNA interaction. This biological regulation can potentially be used to manipulate TLK function to sensitize cells to radiation and chemotherapeutic agents by targeting the DNA damage response pathway.

## Supplementary Material

gkae1279_Supplemental_Files

## Data Availability

The proteomic data for this study have been uploaded to ProteomeXchange, project accession numbers: PXD047964, PXD047966, PXD047996 and PXD048215.
