# Supplementary material for: Autophosphorylation of the Tousled-like kinases TLK1 and TLK2 regulates recruitment to damaged chromatin via PCNA interaction
Source: Nucleic Acids Res. 2024 Dec 27;53(4):gkae1279. doi: 10.1093/nar/gkae1279 (PMC11879137; doi:10.1093/nar/gkae1279)

SUPPLEMENTARY FIGURE LEGENDS

Supplementary Figure 1. Tousled-like kinases 1 and 2 interact with LC8 through an N-terminal LC8 binding motif

(A) Proteomic analyses using SFB-tagged-TLK1 or SFB-TLK2 as baits to identify LC8 and TLKs as their major interacting proteins.

(B, C) LC8 interacts with TLK1 and TLK2 via its binding groove. Cells were transfected with SFB-LC8 or its canonical binding defective mutant (FGSYV/AAAAA), harvested, pulled down using streptavidin beads, and analyzed by Western blot with indicated antibodies.

(D) Schematics of TLK1 and 2 domain organization shows NLS (Nuclear Localization Sequence), LC8 binding motifs IQT for TLK1 and TQS for TLK2, three coiled-coil domains, and serine/threonine kinase domain.

(E) Sequence alignment across species of evolutionarily conserved LC8 binding motifs within the N-termini of TLK1 and TLK2

(F-G) TLK1 and TLK2 interact with LC8 specifically. Cells were co-transfected with SFB-LC8 and TLK1, TLK2 wildtype or LC8 binding defective mutants TLK1 IQT/AAA or TLK2 TQS/AAA, pulled down and analyzed by Western blot with indicated antibodies.

(H) LC8 and TLK1/2 interact directly. In vitro purified MBP-LC8, N-terminal fragment of GST-TLK1 1-243, and GST-TLK2 1-222. GST pulldown was analyzed by Western blot using indicated antibodies.

Supplementary Figure 2. TLK1 and 2 are dimerized and autophosphorylated (A-C). HEK293T cells were transfected with SFB-TLK1 D607A **(A, B)** or SFB-TLK2 D613A **(C)** and the indicated GFP-wildtype and mutants. Pulldown assays using streptavidin beads followed by Western blotting analysis with indicated antibodies.

Supplementary Figure 3. LC8 does not affect TLK1 and TLK2 recruitment to DNA damage sites

(A) Representative images of GFP-TLK1 at 10 min after laser-induced micro-irradiation in U2OS LC8 knockout cells. Scale bar represents 10 µm.

(B) Quantification of the GFP-TLK1 accumulation at the damage site at 10 min after laser-induced micro-irradiation as in A. N=10.

(C) Representative images of GFP-TLK1 D607 mutant at 10 min after laser-induced micro-irradiation in U2OS LC8 knockout cells. Scale bar represents 10 µm.

(D) Quantification of the GFP-TLK1 D607 accumulation at the damage site at 10 min after laser-induced micro-irradiation as in C. N=10.

(E) Representative images of GFP-TLK2 at 10 min after laser-induced micro-irradiation in U2OS LC8 knockout cells. Scale bar represents 10 µm.

(F) Quantification of the GFP-TLK2 accumulation at the damage site at 10 min after laser-induced micro-irradiation as in E. N=10.

(G) Representative images of GFP-TLK2 D613 mutant at 10 min after laser-induced micro-irradiation in U2OS LC8 knockout cells. Scale bar represents 10 µm.

(H) Quantification of the GFP-TLK1 D613 accumulation at the damage site at 10 min after laser-induced micro-irradiation as in G. N=10. Significance was determined by unpaired, two-tailed Student's T-test and P-values are reported as * < 0.05, ** <0.01, *** < 0.001, **** < 0.0001.

Supplementary Figure 4. Regulation of TLK1 at laser-induced DNA damage

(A) Representative images of GFP-TLK1 and D607A recruitment at laser-induced micro-irradiation for 60 min. Scale bar represents 10 μm.

(B) Quantification of the GFP-TLK1 and D607A recruitment as in **(A)**. N=10

(C) Representative images of GFP-TLK1 D607A with endogenous cyclin A staining. Scale bar represents 10 μm.

(D) Quantification of GFP-TLK1 D607A at laser-induced micro-irradiation as in **(C)**. N=13. Significance was determined by unpaired, two-tailed Student's T-test and P-values are reported as * < 0.05, ** <0.01, *** < 0.001, **** < 0.0001.

Supplementary Figure 5. The effects of PI3K-related kinases on TLK1 DNA damage recruitment

(A) U2OS cells were treated for 1 hour with DMSO, 10 μM ATRi (VE-821), 10 μM ATMi (KU55933), or 2 μM DNA-PKi (NU7441) then subjected to 2 Gy irradiation then harvested for Western blot after 1 hour.

(B) SFB-TLK1 wildtype, 9S/T to A, or D607A were treated with λ protein phosphatase and the mobility were analyzed by Western blot.

(C) Representative images of U2OS cells expressing GFP-TLK1 D607A treated with the indicated kinase inhibitor for 1 hour prior to laser-induced micro-irradiation. Scale bar represents 10 μm.

(D) Quantification of GFP-TLK1 D607A accumulation at the damage site after laser-induced micro-irradiation as in **(C)**. N=10.

(E) Representative images of U2OS cells expressing GFP-TLK2 D613A treated with the indicated kinase inhibitor for 1 hour prior to laser-induced micro-irradiation. Scale bar represents 10 μm.

(F) Quantification of GFP-TLK2 D613A accumulation at the damage site after laser-induced micro-irradiation as in **(E)**. N=10. Significance was determined by unpaired, two-tailed Student's T-test and P-values are reported as * < 0.05, ** <0.01, *** < 0.001, **** < 0.0001.

Supplementary Figure 6. TLK1 conserved region a.a. 133-204 is required for its accumulation at damaged chromatin

(A) Schematic illustration of the TLK1 N-terminal deletion fragments.

(B) Expression of the TLK1 N-terminal deletion fragments was analyzed by Western blot.

(C) Representative images of the TLK1 N-terminal deletion fragments illustrated **(A)** at 10 mins after laser-induced micro-irradiation. Scale bar represents 10 μm.

(D) Quantification of the TLK1 N-terminal deletion fragment mutants as in **(C)**. N≥10

(E) Schematic illustration of the full-length TLK1 N-terminal deletion fragments.

(F) Expression of the full-length TLK1 N-terminal deletion fragments was analyzed by Western blot.

(G) Representative images of the lengthTLK1 N-terminal deletion fragments illustrated in **(E)** at 10 mins after laser-induced micro-irradiation. Scale bar represents 10 μm.

(H) Quantification of the full-length TLK1 N-terminal deletion fragments as in **(G)**. N=10. Significance was determined by unpaired, two-tailed Student's T-test and P-values are reported as * < 0.05, ** <0.01, *** < 0.001, **** < 0.0001.

Supplementary Figure 7. PCNA interacts with TLK and regulates their recruitment to DNA damage

(A) Proteomic analysis using TLK1 small fragment (a.a. 133-208) containing the PIP-box sequence.

(B) Representative images of TLK1 a.a. 133-208 fragment and PCNA binding defective mutant (Y149A/F150A) at 10 mins after laser-induced micro-irradiation.

(C) Quantification of the TLK1 fragment accumulation at DNA damage at 10 mins after laser-induced micro-irradiation.

(D) Representative images of GFP-TLK1 D607A in PCNA-depleted cells.

(E) Quantification of GFP-TLK1 D607A accumulation at DNA damage after laser-induced micro-irradiation. N≥ 8.

(G) Representative images of GFP-TLK1 D613A in PCNA-depleted cells.

(H) Quantification of GFP-TLK1 D607A accumulation at DNA damage after laser-induced micro-irradiation. N≥ 10. Significance was determined by unpaired, two-tailed Student's T-test and P-values are reported as * < 0.05, ** <0.01, *** < 0.001, **** < 0.0001.

Supplementary Figure 8. TLK1 PIP-box regulation

(**A**) HEK293T cells transfected with SFB-TLK1 wildtype, D607A, or autophosphorylation 9S/T to A mutant were used for pull down assays using streptavidin beads followed by Western blot analysis with indicated antibodies.

(**B**) HEK293T cells transfected with SFB-TLK1 wildtype, ∆N-terminus, or PCNA binding defective Y149AF150 mutant were used for pull down assays using streptavidin beads followed by Western blot analysis with indicated antibodies.

Supplementary Figure 1

A

Identified Protein	Spectral Counts
TLK1 (Bait)	645
TLK2	194
PGAM5	26
HSPA1B	22
HSPA8	20
HSPA5	16
LC8	15
HNRNPM	14
RPL11	14
ACACA	12

Identified Protein	Spectral Counts
TLK2 (Bait)	1296
TLK1	251
TUBB	114
HSPA8	91
HSPA1B	48
HSPA5	43
LC8	41
ACACA	30
MCCC2	29
HNRNPM	23

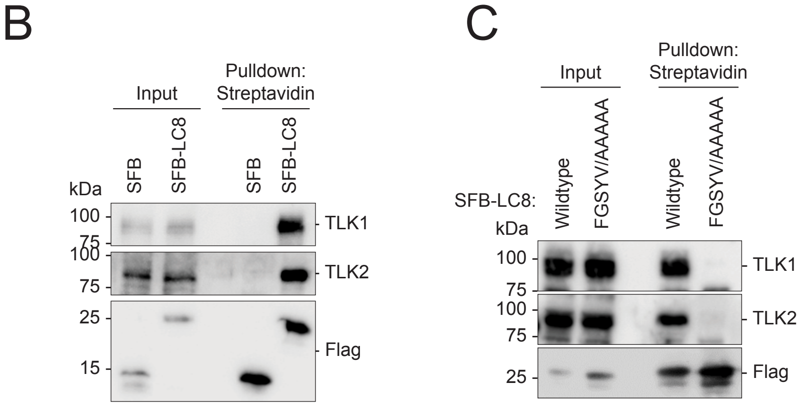

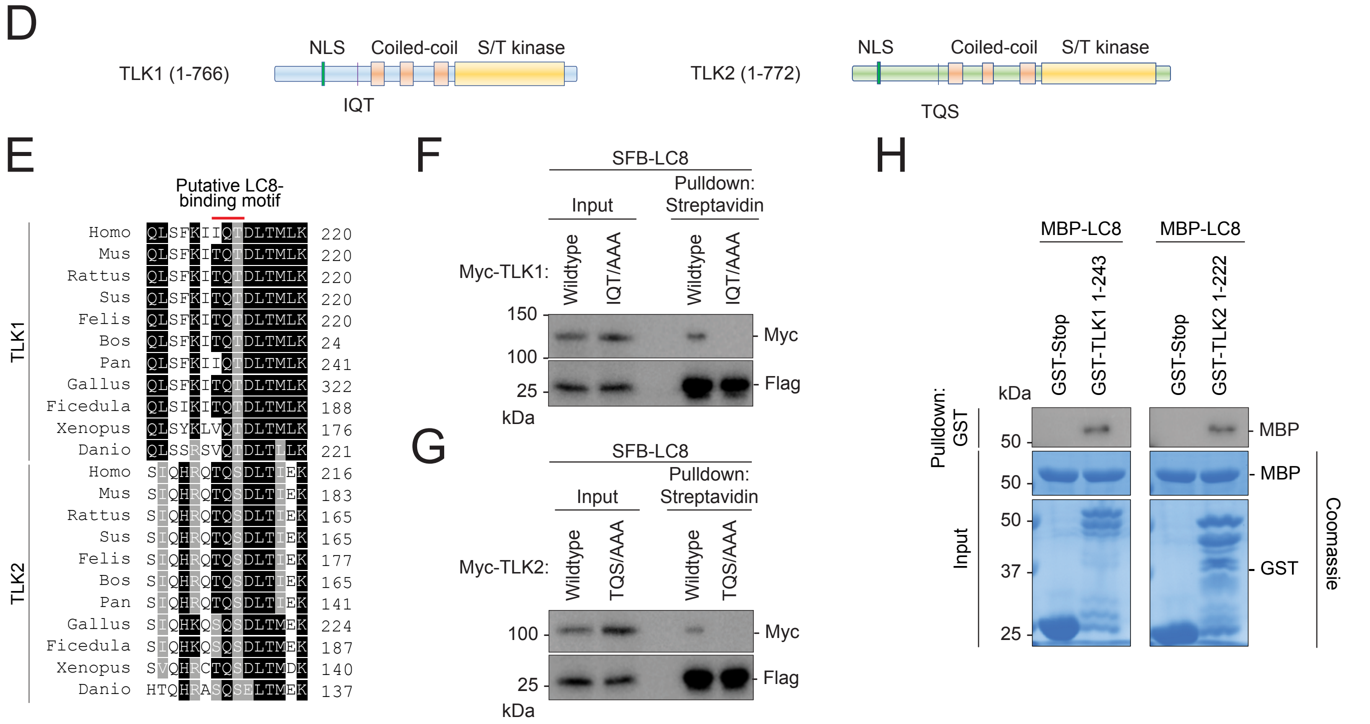

Supplementary Figure 2

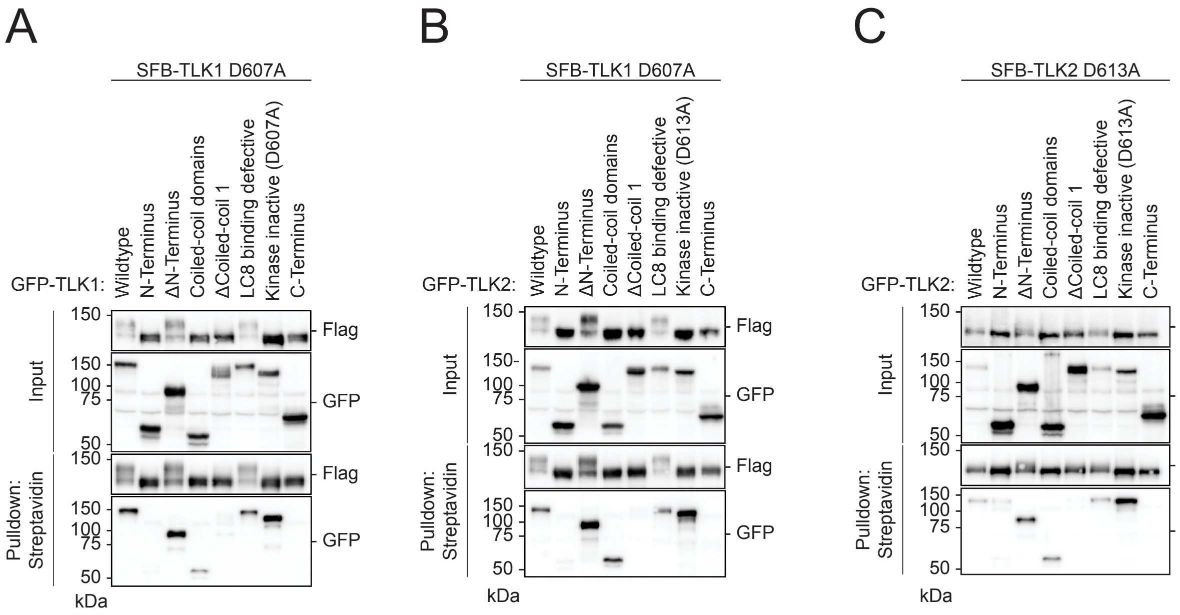

Supplementary Figure 3

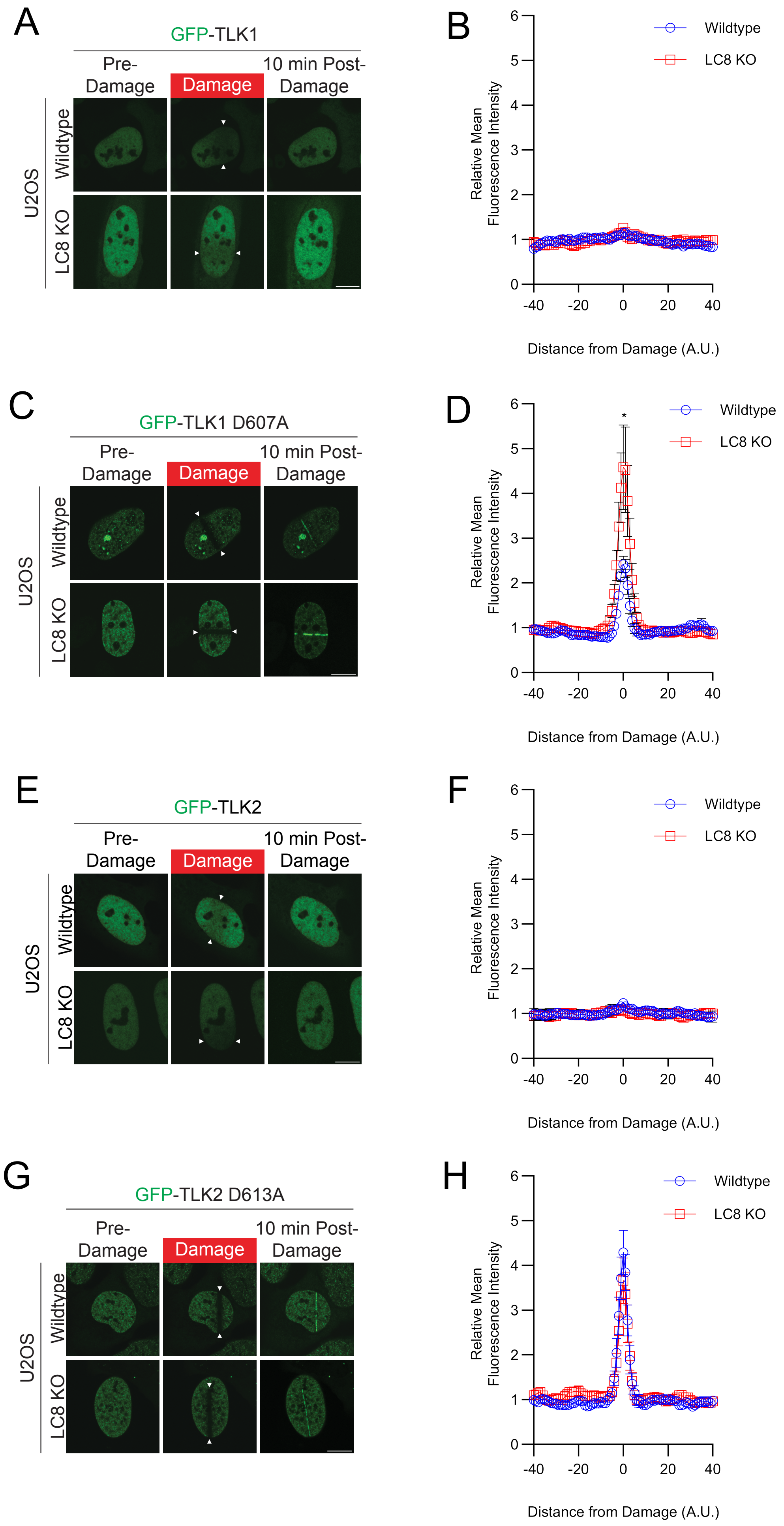

Supplementary Figure 4

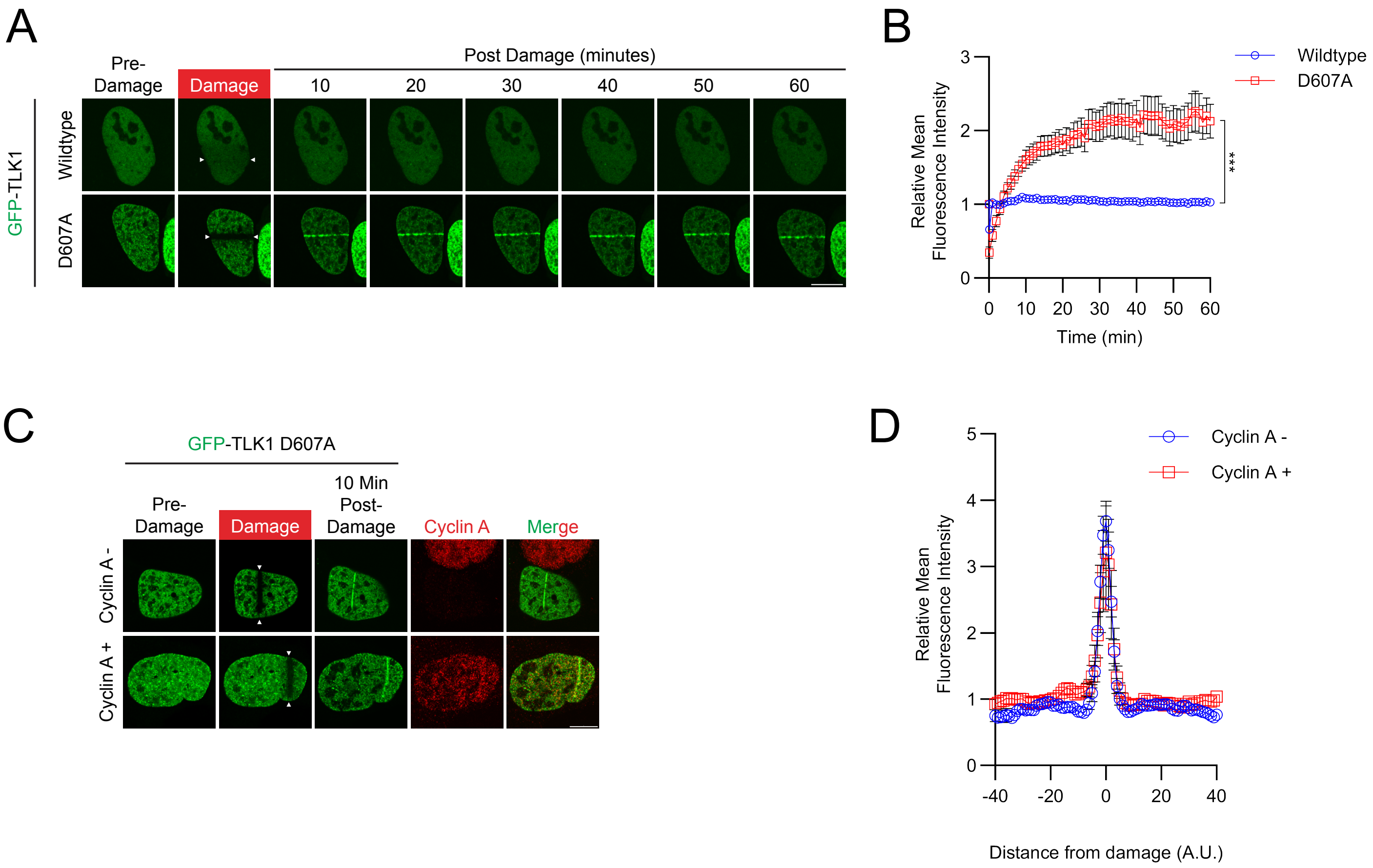

Supplementary Figure 5

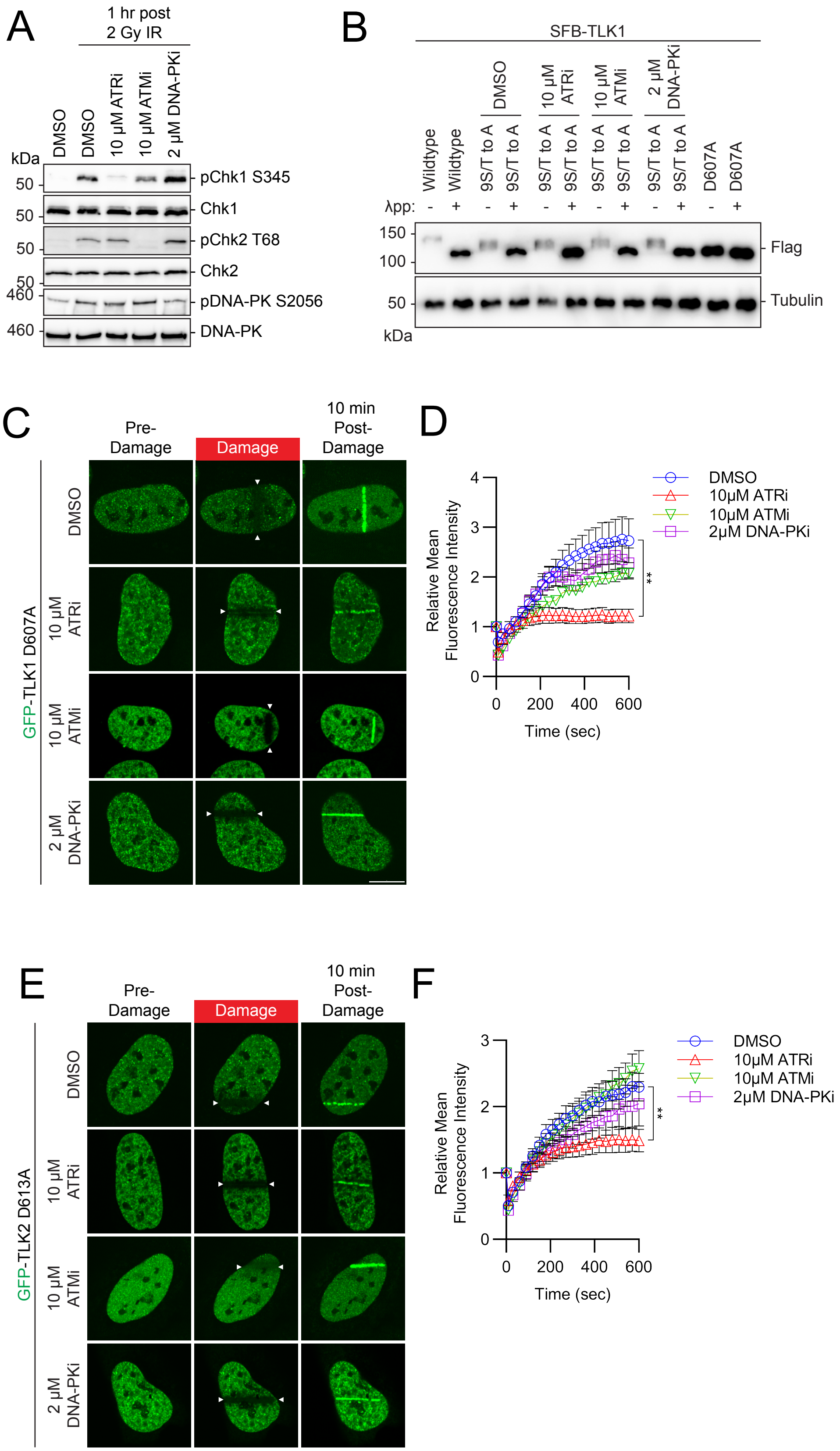

Supplementary Figure 6

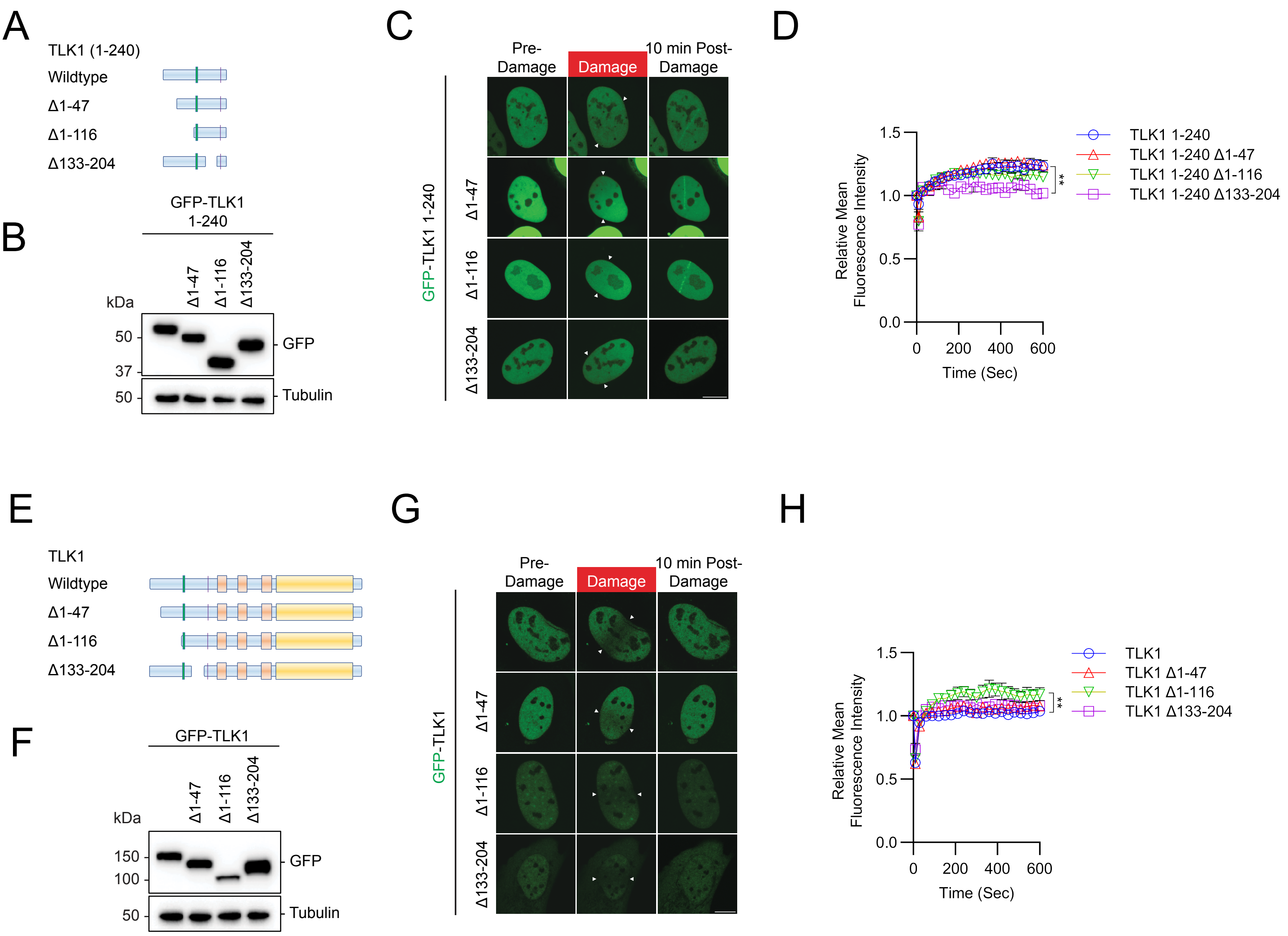

Supplemental Figure 7

A

	Spectral Counts	
Identified Protein	TLK1 133 - 208	TLK1 133-208 Y149A F150A
TLK1(Bait)	19	17
DSP	9	N.C.
HSPA4	7	6
HSPA4L	7	7
RNH1	6	5
PCNA	6	N.C.

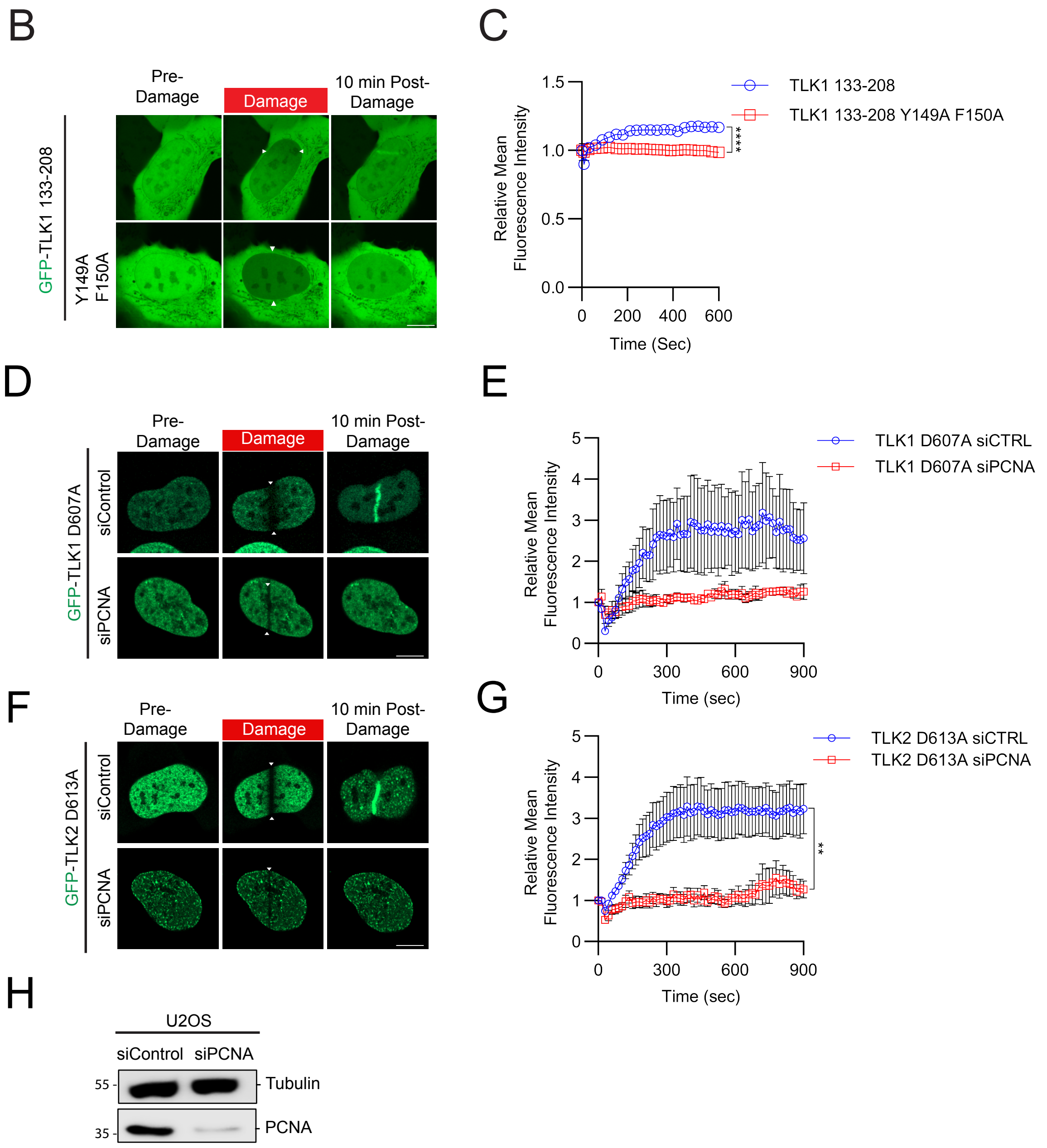

Supplementary Figure 8

A

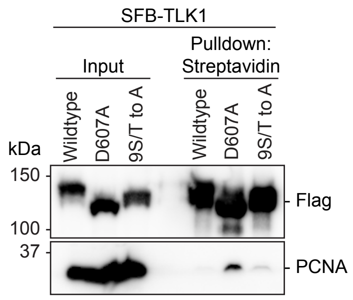

B

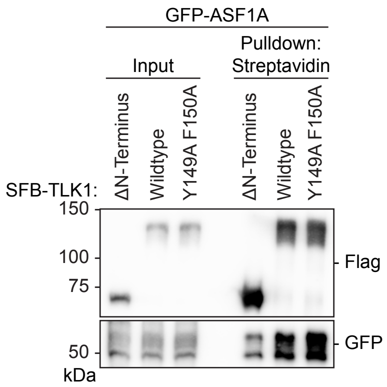

Supplement: gkae1279_Supplemental_Files [file gkae1279_supplemental_files.zip › gkae1279_Supplemental_File.pdf]
