# Supplementary material for: Autophosphorylation of the Tousled-like kinases TLK1 and TLK2 regulates recruitment to damaged chromatin via PCNA interaction
Source: Nucleic Acids Res. 2024 Dec 27;53(4):gkae1279. doi: 10.1093/nar/gkae1279 (PMC11879137; doi:10.1093/nar/gkae1279)
Supplement: gkae1279_Supplemental_Files [file gkae1279_supplemental_files.zip › Supple_Table_1.pdf]

Supplementary Table 1. Primers used for

	Primers: 5’ -> 3’
TLK1 1-243	GGGGACAAGTTTGTACAAAAAAGCAGGCTTC ATGAGTGTCCAAAGTAGCAGT
	GGGGACCACTTTGTACAAGAAAGCTGGGTC CTAGAGCAAATCATCTATACGTCC
TLK2 1-222	GGGGACAAGTTTGTACAAAAAAGCAGGCTTC ATGATGGAAGAATTGCATAG
	GGGGACCACTTTGTACAAGAAAGCTGGGTC ACTGTTTTCTAGTGCAGATA
TLK1 244-766	GGGGACAAGTTTGTACAAAAAAGCAGGCTTC AGGGCTAACTGTGATCTCAGA
	GGGGACCACTTTGTACAAGAAAGCTGGGTC CTAGTAAGTAATTATGCTTGA
TLK2 224-772	GGGGACAAGTTTGTACAAAAAAGCAGGCTTC AATTCTGACTTAGAGAAGAA
	GGGGACCACTTTGTACAAGAAAGCTGGGTC CTAATTAGAAGAACTGTTATTGG
TLK1 244-444	GGGGACAAGTTTGTACAAAAAAGCAGGCTTC AGGGCTAACTGTGATCTCAGA
	GGGGACCACTTTGTACAAGAAAGCTGGGTC CTATGAATTATCTTCATTGTTTAT
TLK2 224-454	GGGGACAAGTTTGTACAAAAAAGCAGGCTTC AATTCTGACTTAGAGAAGAA
	GGGGACCACTTTGTACAAGAAAGCTGGGTC CTAATGATCTTTAAATTGTGAAT
TLK1 ΔCC1	CTTATTGAAAAGAGTACACAAG
	GAGCAAATCATCTATACGTC
TLK2 ΔCC1	AGCAAGAAACTCCTTATAG
	TCCCTCCTTCTTCTCTAAG
TLK1 IQT/3A	GGCTGATCTCACAATGCTGAAATTAG
	GCAGCAATTTTAAAGGATAATTGCTTTG
TLK2 TQS/3A	GGCCGACCTCACAATAGAAAAAATATCTG
	GCGGCCTGTCTGTGCTGGATGGA
TLK1 D607A	CAAAATCACTGCTTTTGGTCTGTC
	ATTTCACCACATGCTGTTC
TLK2 D613A	AAAAATTACAGCTTTTGGTCTTTCGAAG
	ATCTCTCCACACGCTGTA
TLK1 451-766	GGGGACAAGTTTGTACAAAAAAGCAGGCTTC CAGTTCAAAGATCACCCAACA
	GGGGACCACTTTGTACAAGAAAGCTGGGTC CTAGTAAGTAATTATGCTTGA
TLK2 455-772	GGGGACAAGTTTGTACAAAAAAGCAGGCTTC CCAACGCTAAATGACAGATA
	GGGGACCACTTTGTACAAGAAAGCTGGGTC CTAATTAGAAGAACTGTTATTGG
**Primers for Sequential alanine mutations of phosphosites**	
TLK1 S33A	GGCGGCCAGGGCCCTGCTGAATC
	GCCGCCGAGCCCGGGGTT
TLK1 S38 T41/2A	CCAGCTGGGAGGCCCAGGGAAGGT
	CGGCGCGTGATTCAGCAGGGACCTGGC
TLK1 S77A	GAGCACTGGAGCTACGGGCAGTTG

	CCACTTGCAACTCCAGTA
TLK1 S133 S134 S135/3A	GGAAAAGCTATTGGGGGACGTGGCCAC
	CTGAGCAGCTTCATTCTGGTTTTCTGCTTTTCTCTTTCTTC
TLK1 S158 S159/2A	TGGAAATGGCGCAGCtCCAGTAAGAGGC
	CCCTGGTATTCAAAATAGTC
TLK1 S33 S38 T41 S77 S133 S134 S135 S158 S159 /9D gene block	GACCTGCTGAATCACGACCCGCCAGACGGGAGGCCCAGGGA<br>AGGTGCAATGGATGAGCTTCATAGTCTGGATCCAAGAAGGCAA<br>GAGTTATTGGAAGCTAGATTTACTGGAGTTGCAAGTGGGAGCA<br>CTGGAGATACGGGCAGTTGCAGTGTTGGAGCTAAAGCCTCAA<br>CAAATAACGAAAGCTCTAATCACAGTTTTGGAAGCTTGGGATC<br>TTTAAGTGACAAAGAATCAGAGACACCGGAGAAGAAACAATCG<br>GAATCATCCAGGGGAAGAAAGAGAAAAGCAGAAAACCAGAAT<br>GAAGATGACCAGGGAAAAGATATTGGGGGACGTGGCCACAAA<br>ATTAGCGACTATTTTGAATACCAGGGTGGAAATGGCGATGAC
TLK1 pDONR221 linearization	CCAGTAAGAGGCATACCTCCTGCAATCCGTTCTCCTCAAAATT<br>CACATTCACATTCC
	CCTGGCCGCCGCCGCCGA
TLK1 Gene block amplification	GCTCGGCGGCGGCGGCCAGGGAACCTGCTGAATCACGAC
	GGAGGTATGCCTCTTACTGGGTCATCGCCATTTCCACC
**Gibson Assembly primers for domain swapping**	
TLK1_backbone	CAGGACCTGGAAAAGAAGG
	GGTGGAGCCTGCTTTTTTG
TLK2 Nterm	ACAAAAAAGCAGGCTCCACCATGGAAGAATTGCATAGC
	TCCTTCTTTTCCAGGTCCTGCTTACTGTTTTCTAGTGCAG
TLK2 Backbone	AATTCTGACTTAGAGAAGAAG
	GCCAACTTTTTTGTACAAAG
TLK1 Nterm	CTTTGTACAAAAAAGTTGGCATGAGTGTCCAAAGTAGC
	TTCTTCTCTAAGTCAGAATTGAGCAAATCATCTATACGTC
**Primers for N-term deletions**	
TLK1 Δ1-47	GGGGACAAGTTTGTACAAAAAAGCAGGCTTC<br>GCAATGGATGAGCTTCATAG
	GGGGACCACTTTGTACAAGAAAGCTGGGTC<br>CTAGTAAGTAATTAGCTTGA
TLK1 Δ1-116	GGGGACAAGTTTGTACAAAAAAGCAGGCTTC<br>TCGGAATCATCCAGGGGAAG
	GGGGACCACTTTGTACAAGAAAGCTGGGTC<br>CTAGTAAGTAATTATGCTTGA
TLK1 Δ133-204	AAGCAATTATCCTTTAAAATTATTC
	ACTTTCATTCTGGTTTTCTG
**Primers for PIP box mutations**	
TLK1 Y149A F150A	AATTAGCGACGCCGCTGAATACCAGGGTGGAAATGGCTCAAG
	TTGTGGCCACGTCCCCCA
TLK2 Y87A F88A	AATTAGTGATGCCGCTGAGTTTGCTGGGGGAAG
	TTATGTCCCCTAGGAGTG
TLK1 133-208	GGGGACAAGTTTGTACAAAAAAGCAGGCTTC<br>AGTAGTCAGGGAAAAAGTAT
	GGGGACCACTTTGTACAAGAAAGCTGGGTC<br>CTAGGATAATTGCTTTGGTTGTA
