# Supplementary material for: Autophosphorylation of the Tousled-like kinases TLK1 and TLK2 regulates recruitment to damaged chromatin via PCNA interaction
Source: Nucleic Acids Res. 2024 Dec 27;53(4):gkae1279. doi: 10.1093/nar/gkae1279 (PMC11879137; doi:10.1093/nar/gkae1279)
Supplement: gkae1279_Supplemental_Files [file gkae1279_supplemental_files.zip › Supple_Table_2.pdf]

Supplementary Table 2. Phosphorylated peptide identified from DNA repair proteins enriched in HEK293T cells with TLK1 overexpression vs TLK1 and TLK2 depletion

Protein Name	Amino Acid	Position	Adjusted P-value	Log_2 Fold Change
ALKBH5	S	374	6.06E-04	1.64
APTX	S	67	4.17E-05	1.17
ATM	S	1891	1.67E-04	1.40
ATM	S	2996	3.08E-05	1.21
BABAM1	S	57	2.38E-04	1.31
BABAM1	S	62	6.18E-05	1.78
BIVM-ERCC5	S	836	1.84E-03	1.88
BIVM-ERCC5	S	838	1.84E-03	1.88
BRCA1	S	1524	6.99E-05	1.08
DHX9	S	1033	4.96E-03	1.45
EGFR	Y	1197	4.82E-04	1.08
ENDOV	S	106	1.12E-04	1.40
EYA4	S	4	6.14E-03	2.19
EYA4	S	24	9.22E-03	1.83
EYA4	S	27	6.13E-03	1.97
FANCA	S	189	3.73E-05	2.75
FANCD2	S	592	1.75E-04	1.48
GINS2	S	179	3.12E-02	2.53
GINS2	T	180	8.40E-04	1.72
GINS2	S	182	8.40E-04	1.72
GPS1	S	463	5.32E-04	1.32
HMGA1	S	9	1.37E-04	1.27
HMGB1	S	35	2.74E-03	1.25
HUWE1	S	2940	3.25E-03	1.54
HUWE1	S	3757	4.82E-03	1.06
HUWE1	S	3760	6.97E-03	1.10
HUWE1	S	3799	5.56E-04	2.85
HUWE1	S	3919	1.54E-02	2.23
MDC1	S	108	5.61E-05	1.16
MDC1	S	299	1.30E-02	1.42
MDC1	T	301	1.30E-02	1.42
MDC1	S	307	1.30E-02	1.42
MDC1	T	652	2.05E-06	3.44
MDC1	T	654	2.05E-06	3.44
MDC1	S	995	2.47E-03	1.23
MDC1	S	1086	1.66E-03	1.03
MDC1	S	1540	2.32E-04	3.60
MGME1	S	71	1.15E-03	2.27
MGME1	S	343	6.22E-05	1.01
MLH1	S	374	9.50E-03	1.19

Protein Name	Amino Acid	Position	Adjusted P-value	Log_2 Fold Change
MLH1	S	446	6.15E-05	1.40
NSMCE3	S	57	1.92E-05	2.13
NSMCE3	S	60	1.92E-05	2.13
NUCKS1	S	19	7.74E-05	1.26
NUCKS1	S	30	7.49E-03	1.46
NUCKS1	S	50	8.37E-03	1.06
NUCKS1	S	54	1.01E-04	1.41
NUCKS1	S	58	1.01E-04	1.41
NUCKS1	S	61	1.01E-04	1.41
NUCKS	S	73	3.89E-03	1.35
NUCKS	S	75	3.89E-03	1.35
NUCKS	S	79	3.89E-03	1.35
NUCKS1	T	202	4.19E-04	1.39
NUCKS1	S	204	1.55E-04	1.70
PARP1	S	179	4.18E-02	2.17
PARP1	S	785	1.37E-03	1.10
PDS5A	S	1182	1.79E-02	1.95
PML	S	403	8.92E-05	1.51
PML	S	408	8.92E-05	1.51
POLE	S	20	3.96E-06	3.35
PRKDC	S	2624	3.12E-02	1.42
PRKDC	S	2626	6.74E-06	2.74
PRKDC	Y	3168	1.95E-04	1.85
PRKDC	S	3205	5.59E-06	1.94
RAD21	S	545	7.83E-04	1.32
RAD23B	S	160	8.05E-04	2.19
RAD23B	S	168	3.78E-05	3.55
RFC1	S	190	4.80E-05	1.26
RFC1	T	193	1.18E-02	1.02
RFC1	S	564	2.02E-04	3.37
SMARCAD1	S	986	5.89E-06	1.73
SMC1A	S	336	1.31E-05	2.00
SMC1A	S	338	1.31E-05	2.00
SMC1A	S	934	1.66E-05	3.72
SMC1A	S	940	8.14E-03	1.17
SPIDR	S	18	4.38E-04	1.18
TCEA1	S	209	2.34E-03	1.12
TNKS1BP1	S	437	4.18E-04	2.78
TNKS1BP1	S	601	2.59E-04	1.14
TNKS1BP1	S	899	8.55E-04	2.30
TNKS1BP1	S	983	3.34E-02	1.32
TNKS1BP1	S	1008	5.02E-03	1.87

Protein Name	Amino Acid	Position	Adjusted P-value	Log_2 Fold Change
TNKS1BP1	S	1328	1.10E-02	1.05
TNKS1BP1	S	1331	1.10E-02	1.05
TP53	S	15	1.14E-04	1.80
TP53	T	18	1.10E-04	1.55
TP53	S	302	1.37E-04	2.04
TP53	S	304	1.37E-04	2.04
TP53BP1	S	83	1.01E-04	1.64
TP53BP1	S	227	6.15E-05	2.57
TP53BP1	T	307	9.34E-03	1.24
TP53BP1	S	528	8.40E-05	1.61
TP53BP1	S	530	8.40E-05	1.61
TP53BP1	T	548	7.35E-03	2.07
TP53BP1	S	557	7.35E-03	2.07
TP53BP1	S	814	7.38E-04	2.62
TP53BP1	S	998	1.57E-02	1.07
TP53BP1	T	1001	1.57E-02	1.07
TRIM28	S	26	1.70E-04	1.54
TRIM28	S	437	1.59E-03	2.47
TRIM28	T	541	7.33E-03	1.03
TRIM28	S	681	7.30E-06	2.65
TRIM28	S	683	1.14E-02	1.96
TRIM28	S	828	1.45E-03	2.67
UBE2V1	S	146	7.80E-04	1.10
UIMC1	S	101	5.09E-04	1.48
UIMC1	S	463	2.38E-05	2.20
UIMC1	S	466	2.38E-05	2.20
UPF1	S	1107	3.06E-05	1.29
VCP	S	784	4.51E-05	2.23
VCP	S	794	3.96E-06	2.27
VCP	Y	805	8.64E-03	2.52
WDHD1	S	383	3.78E-05	1.70
WDHD1	S	853	1.16E-02	1.06
WDR70	S	638	6.78E-04	1.22
XAB2	S	851	1.76E-05	2.77
XPC	S	94	6.14E-03	1.26
XPC	S	883	1.19E-02	2.50
XPC	S	884	1.19E-02	2.50
XPC	S	891	1.28E-04	2.21
XRCC4	S	260	9.86E-05	2.12
XRCC4	S	299	1.38E-03	3.36
XRCC4	S	304	3.89E-03	1.39
XRCC6	S	2	6.50E-06	3.47

Protein Name	Amino Acid	Position	Adjusted P-value	Log_2 Fold Change
XRCC6	S	27	7.30E-06	1.79
XRCC6	S	560	1.95E-04	1.11
